# Recycling and Recovery of Cathode Materials from Spent Lithium-Ion Batteries by Hydrometallurgy: A Review

**DOI:** 10.3390/ma19194242

**Published:** 2026-10-07

**Authors:** Alpha Chi Him Tsang, Jing Ji, Dawson Wai-Shun Suen, Chi-Wing Tsang, Vanessa Chi Ying Li, Xiao-Ying Lu

**Affiliations:** Department of Construction, Environment and Engineering, Technological and Higher Education Institute of Hong Kong, 20A, Tsing Yi Road, Tsing Yi, Hong Kong, China

**Keywords:** cathode materials, hydrometallurgy, leaching kinetics, extraction methods, co-precipitation methods

## Abstract

With the popularization of electric vehicles, lithium-ion batteries (LIBs) have begun to be used in large quantities, and the scale of the waste batteries is large and growing rapidly. So, the environmental pressure faced and the economic benefits contained in waste batteries are rapidly increasing every year. The valuable metals (e.g., Ni, Co and Mn) in the cathodes of LIBs are important non-renewable resources with high recovery value. So, the recovery and reuse of valuable metals in waste LIBs have constituted an important issue of concern. This review focuses on the hydrometallurgy of spent-LIB cathode, the extraction of valuable metals (solvent extraction and filtration), and co-precipitation methods. A perspective and outlook associated with the challenges and limitations of current hydrometallurgy research is proposed for breakthrough development of such technology for future industrialization.

## 1. Introduction

Recently, with the vigorous development of new energy vehicles, LIBs production has grown rapidly due to the excellent performance of these batteries and the necessity for a low-carbon economy [1]. Currently, they are the most used battery types in mobile electronics [2]. According to the global electric vehicle (EV) outlook of the International Energy Agency [3], EV sales in 2021 doubled from the previous years to a new record of 6.6 million. However, due to the internal redox reaction of the battery, its average service life is about 7~8 years, which means that a large number of waste batteries will have reached the end of their service life in the future and will need to be disposed of properly. The production of LIBs consumes tons of non-renewable elements, such as lithium (Li), nickel (Ni), cobalt (Co), and manganese (Mn), which will cause great harm to the environment and the human body if not handled properly. For example, excessive cobalt in the human body will cause hearing and vision damage and lithium can cause damage to the body’s central nervous system and kidneys [4,5]. In addition, China’s cobalt and nickel resources are scarce, and these reserves only account for 1% and 3.4% of the world’s total. So, the use of these materials is limited and expensive. However, the cost of cobalt is relatively low (~€26.6 per kg) and it is regarded as sustainable metal according to its relatively low global warming potential (8.3 CO_2_-eq per kg Co) from cradle to gate, compared with other valuable metals [6]. As a result, effective recycling can bring considerable economic benefits. For example, the current profit generated by recycling NCM waste batteries is about $1400/t. Therefore, the recycling of LIBs is considered to be an important way to solve the problem of raw material supply for EV production. It can be seen from the perspective of the associated life cycle that the recycling of LIBs has positive benefits of reducing carbon emissions, resource consumption and potential harm to humans [7].

**Figure 1:**
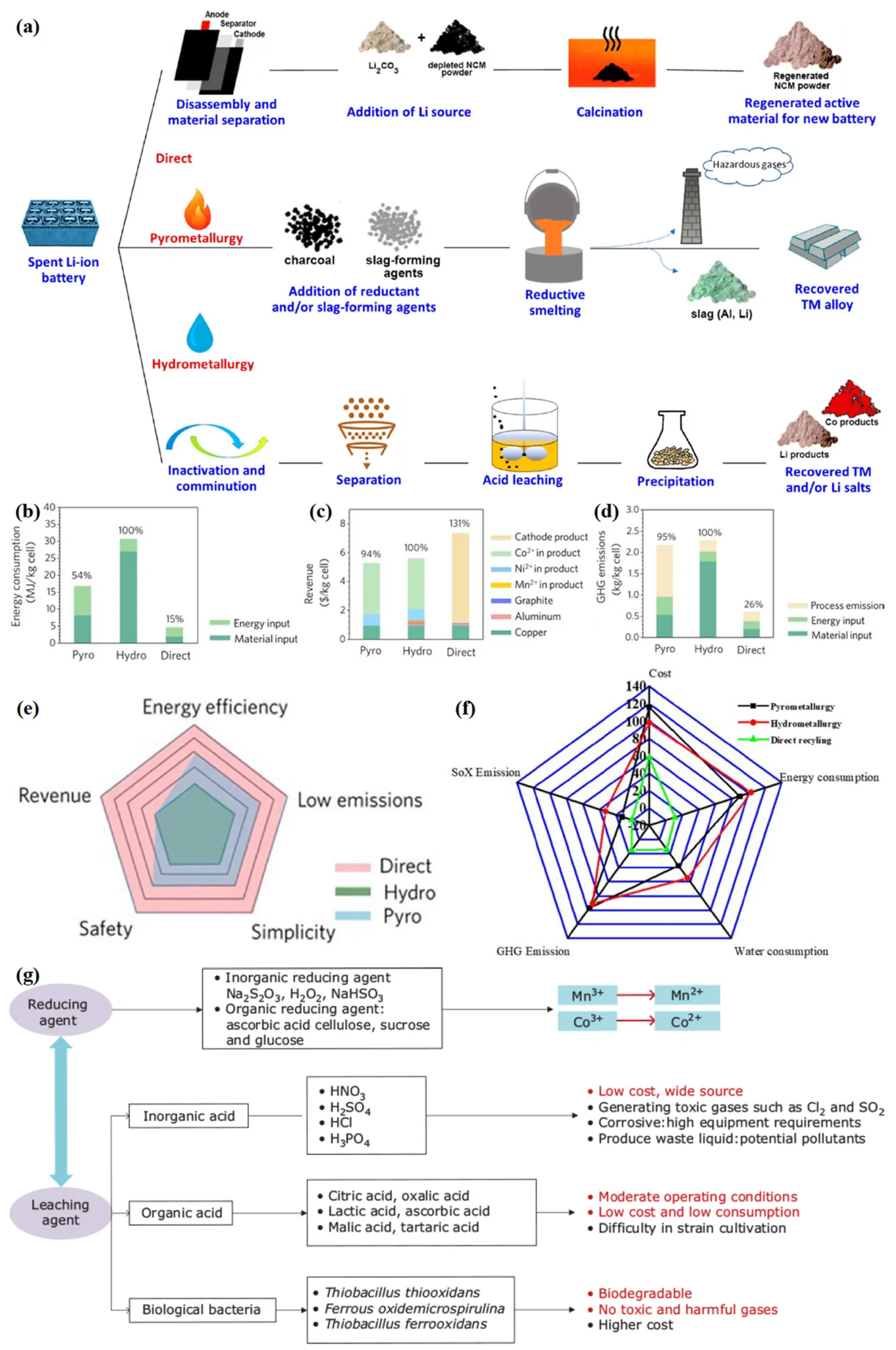
(**a**) Typical direct, pyrometallurgical, and hydrometallurgical recycling methods for recovery of Li-ion battery active materials. From top to bottom, these techniques are used by OnTo, Umicore, and Recupyl in their recycling processes (some steps have been omitted for brevity) [16]. (Reproduced with permission, 2022, ACS). (**b**) Life cycle analysis of pyrometallurgical (Pyro), hydrometallurgical (Hydro), and direct (Direct) recycling methods. Energy consumption (**b**), GHG emissions (**c**), potential revenue from outputs produced (**d**), and spider chart comparing various features (**e**) of Pyro, Hydro, and Direct recycling methods. (**f**) The comparison of the cost, energy consumption, and environmental impacts of producing a 1 kg NMC111 cathode and recycling it with different techniques [17]. (Reproduced with permission, 2022, Wiley VCH-GmbH). (**g**) Separation methods for leaching metals from active materials [10]. (Reproduced with permission, 2021, Johnson Matthey).

At present, power batteries mainly include nickel metal hydride (NiMH) batteries, lithium cobalt oxide (LCO: LiCoO_2_) batteries, lithium iron phosphate (LFP: LiFePO_4_) batteries, lithium manganese oxide (LMO: LiMn_2_O_4_) batteries and ternary polymer LIBs, of which ternary polymer LIBs can be divided into nickel–cobalt–manganese (NCM) and nickel–cobalt–aluminum (NCA) batteries [8]. Due to the high cost and poor safety of LCO batteries, many studies have focused on the replacement of LCO, and have developed batteries that use Ni, Mn, Fe and other cheap transition metals to completely or partially replace Co, such as LiNi_1/3_Co_1/3_Mn_1/3_O_2_, LiNi_0.8_Co_0.15_Al_0.05_O_2_, LMO and other new cathode materials [9]. With the development of NCM batteries, since 2018, the installed capacity of NCM batteries has gradually surpassed that of LFP batteries, and NCM batteries consist of more valuable metals that are worth recycling, compared with traditional LCO and LFP batteries [10]; therefore, the recycling research investigating NCM batteries has also become important. The NCM523 (nickel:cobalt:manganese at a ratio of 5:2:3) is the most used type of NCM battery in China, accounting for nearly 75% of the NCM series, and the NCM 811 accounts for almost a fifth of EV LIB deployment in China [11]. In NCM batteries, the cathode, which is rich in nickel, cobalt and manganese, accounts for a large proportion of the total battery mass. This is particularly significant in the context of scarce mineral resources and rising commodity prices. Hence, it has a very high recycling value. The recycling process of end-of-life LIBs includes pre-treatment, extraction and regeneration [1]. The battery is completely discharged, disassembled and broken, subjected to physical separation or chemical dissolution, and then undergoes secondary treatment to recover electrode materials [1,10]. Existing spent-LIB recycling processes include pyrometallurgy, hydrometallurgy and biometallurgy [12,13,14,15]. Each technology has its own advantages and disadvantages (Figure 1). However, the current commercial recycling technology is mainly based on hydrometallurgy due to the fact that these methods have a small impact on the environment in terms of wet recycling and high purity of recovery, in addition to the possibility of mass production.

**Figure 2:**
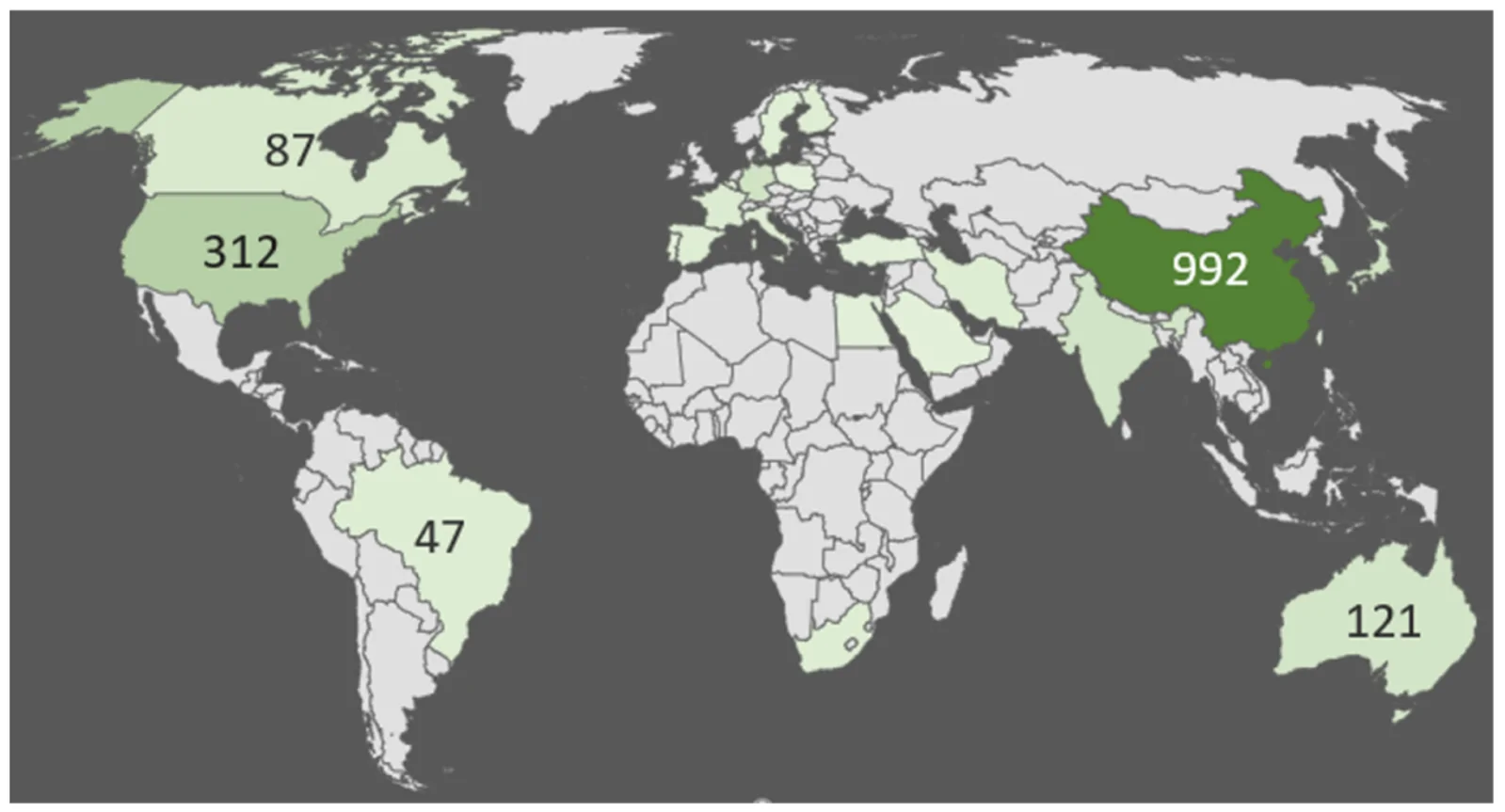
Publications per year–demographic distribution, with the number of publications in the LIB recycling field [18]. (Reproduced with permission, 2022, MDPI).

At present, the recovery of lithium, cobalt, nickel and other elements in scrapped LIBs has become a focus of attention in various countries. For example, the European Union has formulated a series of regulations related to the recycling of power LIBs, such as the Battery Directive and the End-of-life Vehicle Directive. Statistics show that since 2000, there have been more than 2437 non-repeated articles about the recycling of LIBs worldwide, of which 992 articles have been published in China on the recycling and reuse of secondary batteries (green part), accounting for about 40.6% of the total, followed by the United States (pale green part), which accounts for 12.6% (312 publications) (Figure 2) [18]. The articles on the recycling of used batteries have maintained a rapid growth trend since 2010, and more than 500 relevant papers were published in 2022 (Figure S1) [18]. Researchers in South Korea, Germany, India, Brazil and other countries are also publishing articles in the field of secondary battery recycling, a trend which is gradually increasing, and the attention in various other countries has also increased steadily, so there is still a large space for exploration in this field.

In the current review, one major direction of focus is the recycling of valuable metals via the relatively green method of hydrometallurgy; this comprises the first part. The second part of the review examines the post-treatment process, including individual metal extractions and the co-precipitation for battery recycling, in addition to selected industrial application examples. Finally, challenges associated with the current hydrometallurgy technology and the resulting perspective and outlook are also explored.

## 2. Acid Leaching and the Kinetics of Leaching

The hydrometallurgical process is the most primitive and important leaching process for the recovery of metals such as lithium, nickel, cobalt and manganese from spent LIBs. Among the hydrometallurgical techniques for recycling spent LIBs, acid leaching is the most cost-effective, simple and environmentally friendly technique [9,19]. Hydrometallurgy can convert valuable metals from stable cathodic material compounds to forms easily soluble in solution media, thereby providing raw materials for subsequent analyses and purification processes. Hydrometallurgy can be divided into acid leaching and alkali leaching, of which acid leaching can be subdivided into organic acid leaching and mineral acids leaching. The mineral acid leaching reagents are mainly sulfuric acid (H_2_SO_4_), hydrochloric acid (HCl), nitric acid (HNO_3_), phosphoric acid (H_3_PO_4_) and hydrofluoric acid (HF). Some organic acids can be used as a reductant (ascorbic acid and lactic acid), precipitant (oxalic acid, critic acid, etc.) or chelating agent (citric acid, malic acid, succinic acid and aspartic acid) during the cathodic material recovery process [14,20,21,22,23,24,25]. The organic acid with a chelating function refers to a methodology of organic acid in which the valuable metal in the battery cathode is chelated with the functional group of the organic acid during the leaching process in order to be leached efficiently. The organic acid with a reducing function mainly refers to organic acids that can act as a leachant and a reductant simultaneously in the leaching process to recover the valuable metal elements in the cathode materials. The organic acid with precipitation function describes a method in which metal ions and organic anion groups in acid will form a precipitate during the organic acid leaching process.

The efficiency of the acid-leaching process could be influenced by H^+^ concentration, reaction temperature, reaction time, and solid-to-liquid ratio (S/L) [26]. Therefore, in research, single-factor experiments are usually conducted to tentatively explore and determine the appropriate ranges of acid concentration (C, mol/L (M)), agitation rate, retention time (min), reaction temperature (°C), concentration, solid-to-liquid ratio (S/L, g/L) and reductant dosage (*v*/*v* % or g/g) [27]. Although Zhang et al. [28] believed that the influence of the solid-to-liquid ratio (S/L) on leaching is not obvious, in order to ensure the integrity of the experiment, the S/L is generally studied as a variable.

### 2.1. Leaching of Mineral Acids

Sulfuric acid, hydrochloric acid, and nitric acid are the most common leaching agents for acid leaching of spent LIBs, and many studies report on the leaching of valuable metals in the separated cathode materials using those mineral acids with hydrogen peroxide (H_2_O_2_) as the reductant [13,15,29,30,31,32]. This is thanks to the high acidity of these acids (pK_a_ < 0) [33,34,35,36] and due to the fact that they are easy to obtain [37] and relatively low cost [38]. According to the results of mineral acid-driven spent-LIB metal leaching process summarized in Table 1, the data shows that efficiency of metal recovery achieved by hydrochloric acid and sulfuric acid can reach the ranges of 81.00–100% (Li) [33,34,35,36,39,40,41], 93.38–98.70% (Ni) [39,40,41], 91.60–99% (Co) [39,40,41], and 87.90–97.10% (Mn) [39,40,41], with high diversity in the sources of the spent LIBs, including mobile phones, NCM batteries, and LCO batteries [30,33,36,42,43], under optimized conditions. In contrast, the effectiveness of metal recovery was relatively lower when nitric acid (Co: 85% [43]; Co: 40% to 85%, Li: 75% to 85%) [44] or phosphoric acid (Li: 88–99% and Co: >99%) [45,46] were used (Table 1). Some unique strengths of phosphoric acid as a leachant are that (1) Li_3_PO_4_ is the final product, which reduces the number of steps required to obtain lithium phosphate [45]; (2) the method is relatively less corrosive [46]; (3) the method has a relatively lower environmental impact [41]; and (4) the technique is associated with a lower cost, and the materials are easier to obtain.

High metal recovery efficiency was achieved with help of reducing agents among the reported examples (Table 1), of which H_2_O_2_ is the most extensively used [12,43,45,46,47,48,49]. The major role of H_2_O_2_ throughout the acid leaching process is to (1) speed up the dissolution of Co and promote the dissolution of Li [43]; (2) reduce the Co^3+^ to Co^2+^ and Mn^4+^ to Mn^2+^ in the spent LIBs [31,44,50]; and (3) utilize synergistic promotion effects of transition metals such as Co and Ni [51] to enhance redox and leaching kinetics in cathode recycling. While H_2_O_2_ become unstable after prolonged storage [40], alternative inorganic reducing agents include sodium sulfite (Na_2_SO_3_), sodium hydrogen sulfite (NaHSO_3_), and sodium thiosulfate (Na_2_S_2_O_3_); these were used throughout sulfuric acid-driven acid leaching process [12,32,40,52,53]. For example, the efficiency levels of the Li and Co recovery from LCO achieved by the H_2_SO_4_/Na_2_S_2_O_3_ system were higher than 99% [52] and those of Li, Ni, Co and Mn were 96.70%, 96.40%, 91.60% and 87.90% for the laptop spent-LIB mixture by same acid/reducing agent [40], respectively. In addition to the use of inorganic reducing agents, the organic compounds included glucose [31,54], L-ascorbic acid [55], and citric acid [41]; these were used within spent-LIB metal recovery approaches using sulfuric acid [31], nitric acid [55] and phosphoric acid [41], respectively. The corresponding efficiency levels achieved were 85% (Li and Co) [31]; 100% (Li, Ni, Co, and Mn) [55]; and 100% (Li), 93.38% (Ni), 91.63% (Co), and 92.00% (Mn) [41], as summarized in Table 1, respectively. The role of the studied organic agents was to act as chelating agent by capturing metal ions, which speeds up the leaching speed. However, there are exceptional cases which do not require the use of reducing agents [35,36,42,56], such as the demonstration by Li’s team that the metal recovery from spent LIBs (LCO) achieved can be up to 97% (Li) and 99% (Co) under HCl leaching [36] without help of the reducing agent H_2_O_2_. Such results reflect the finding that whether a reducing agent is mandatory for effective metal recycling will be a case-dependent question, especially in the context of moving to industrial-scale operation.

Sometimes mineral acids are also studied in the context of anodic metal recovery. For example, a demonstration by Guo’s team showed that high Li recovery (99.4 wt%) was achieved by hydrochloric acid-driven spent-LIB anode leaching with a reducing agent of H_2_O_2_ and a leaching period of 50–130 min [30]. The performance did not differ too much without the use of H_2_O_2_ (Acid:H_2_O_2_ = 10:1, Li leaching efficiency = 97.1%), under optimized leaching conditions (Table 1) [30]. The HCl-assisted anode leaching is a multiple-step reaction due to the co-existence of water-soluble anodic Li (Li_2_O, Li_2_CO_3_, CH_3_OLi) and water-insoluble anodic Li (ROCO_2_Li and LiF), which results in Li salt hydrolysis (soluble Li) and double decomposition (insoluble Li) at the same time [30,57,58].

**Table 1:** Activities of mineral acid-driven leaching of spent LIBs.

Acid (Concentration (M))	Spent LIBs Target	Reducing Agent (Concentration)	Solid-to-Liquid Ratio (g/L)	Metal Recovery (%)	Ref.
Hydrochloric acid (3 M)	LCO	H_2_O_2_ (3.5% *v*/*v*)	N/A	Li: 90%, Co: 90%	[43]
Hydrochloric acid (4 M)	LCO	N/A	N/A	Li: 97%, Co: 99%	[36]
Hydrochloric acid (3 M)	Li (Anode)	N/A	20 g/L	Li: 97.1%	[30]
Hydrochloric acid (3 M)	Li (Anode)	H_2_O_2_ (2.73% *v*/*v*)	20 g/L	Li: 99.7%	[30]
Sulfuric acid (2 M)	NCM, LCO, LMO mixture	H_2_O_2_ (2% *v*/*v*)	N/A	Li: 81.00%, Ni: 98.70%, Co: 98.20% Mn: 97.10%	[39]
Sulfuric acid (1 M)	NCM	NaHSO_3_ (0.075 M)	20 g/L	Li: 96.70%, Ni: 96.40%, Co: 91.60% Mn: 87.90%	[40]
Sulfuric acid (3 M)	LCO	Na_2_S_2_O_3_ (0.25 M)	N/A	Li: 99%, Co: 99%	[52]
Nitric acid (3 M)	N/A	H_2_O_2_ (1.7 *v*/*v*%)	N/A	Co: 98%	[56]
Nitric acid (0.5 M)	NCM	L-ascorbic acid (0.5 M)	20 g/L	Li: ~100%, Ni: ~100%, Co: ~100%, Mn: ~100%	[55]
Phosphoric acid (0.7 M)	LCO	H_2_O_2_ (4% *v*/*v*)	50 g/L	Li: 88%, Co: >99%	[46]
Phosphoric acid (0.292 M)	LCO	H_2_O_2_ (2% *v*/*v*)	8 g/L	Li: 99%, Co: >99%	[45]
Phosphoric acid (0.2 M)	NCM	Citric acid (0.4 M)	20 g/L	Li: 100%, Ni: 93.38%, Co: 91.63%, Mn: 92.00%	[41]
Phosphoric acid (1.5 M)	LCO	Glucose (0.02 M)	2 g/L	Li: 100%, Co: 98%	[54]

N/A: No reducing agent.

### 2.2. Organic Acid Leaching

The use of inorganic strong acids (mineral acids: pK_a_ < 0), such as HCl, HNO_3_, and H_2_SO_4_, results in the release of a significant amount of toxic gas (Cl_2_, SO_2_, and NO_x_) during leaching, and these gases are a threat to the environment and human health [9,23,59]. In contrast, although the acidity of mild organic acids is weaker (pK_a_ > 0), the secondary pollution is lessened [23,60]. At the same time, the use of an organic acid with high acidity (pK_a_ < 0) was one way to eliminate the adverse effects of secondary pollution due to evolving toxic gases (e.g., Cl_2_, SO_2_, NO_x_, etc.) when achieving high metal recovery efficiency (~100%). Furthermore, some strong organic acids, like methanesulphonic acid (MSA), are claimed to be biodegradable [61], which can make the process green in nature. Without compromising leaching efficiency, there are reports of using mild organic acids such as citric acid, ascorbic acid, malic acid, oxalic acid, aspartic acid, succinic acid, etc. [19,20,60,62,63], and the strong organic acids have included MSA [61], benzenesulphonic acid [59], and p-toluene sulfonic acid (PTSA) [64,65].

#### 2.2.1. Weak Organic Acids

The group of weak organic acids (pK_a_ > 0) includes citric acid, ascorbic acid, lactic acid, tartaric acid, oxalic acid, and malic acid, which are commonly used in the hydrometallurgy of spent LIBs; these have at least two carboxylate groups or hydroxyl groups, which gives them the capacity to dissociate metal ions from the spent-LIB cathode powder and act as a chelating group for effective metal recovery. Another unique advantage of applying weak organic acid for spent-LIB recycling is in their naturally existing properties relative to their low cost, such as with L-ascorbic acid [20,62] and tartaric acid [66,67,68]. Such properties have made weak organic acids relatively green candidates for spent-LIB metal recycling.

The activity of weak-acid-driven spent-LIB metal leaching is summarized in Figures S2–S4 and S5, and Table 2 shows that most of the weak organic acids can achieve high metal recovery percentages up to the ranges of 93–100% for Li [20,22,62,63,69,70] and 91–100% for Co [20,22,23,62,63,69,70] in spent LCO LIBs. Only few demonstrations of weak organic acid were focused on the metal recycling from spent LIBs out of LCO. For example, lactic acid [9], citric acid [27], tartaric acid [66], and oxalic acid [71] have been selected for spent NCM LIB metal recovery, and most of the selected studies achieved high leaching efficiency for Li (97–100%), Ni (89–100%), Co (92–100%), and Mn (94–100%) [9,27,66], respectively. One of the studies focused on the effective recovery of Li from the spent NCM mixture rather than complete metal recovery, which reflected the extremely large difference between Li recovery (75%) and those for the rest of the metals (<1% for Ni, Co, and Mn) [71], as shown in Figure S5 and Table 2. Another demonstration by Li’s research group, on the oxalic acid-driven spent LFP recycling, showed a recovery and separation of Li from Fe which reflected high Li recovery (100%), compared to the very low Fe recovery (8%) in the mixture [72]. One of the organic acid-driven spent-LIB recycling demonstrations involving the spent NCM and use of citric acid as leachant showed the reusability of the citric acid, for five times, where metal recovery percentage was not depreciated too much after five cycles of leaching (first cycle vs. fifth cycle, Li: 99% vs. 97%, Ni: 91% vs. 89%, Co: 92% vs. 92%, Mn: 94% vs. 91%) via reusing the citric acid [27] (Table 2). This finding showed the possibility of leachant recycling throughout the metal recycling when the process is raised to an industrial-scale operation.

Just as with the mineral acid-assisted spent-LIB hydrometallurgy, reducing agents were also beneficial to the metal recovery; H_2_O_2_ was the most widely used reducing agent (Table 1) [9,62,63,66,69]. Some of organic acid-driven leaching reactions use alternatives, including ascorbic acid, in order to make reaction process greener [19,20,62] and utilize chelating with metal ions like Mn^2+^ and reduction of Co^3+^ to Co^2+^ [20]. For example, in the LCO leaching by tartaric acid, with ascorbic acid (AA, 0.02 M) as a reducing agent, the UV-vis spectra of the Co(II)-tartarate solution showed the presence of a Co(II)-tartarate complex absorption peak (λ_max_ = 300 nm) with increasing intensity in the leachate as the leaching time passed (10–300 min). However, both a Co(II)-tartarate complex absorption peak (λ_max_ = 240 nm) and a Co(III)-tartarate complex peak (λ_max_ = 512 nm) were observed when AA was not used. This finding reflected that the AA reduced Co(III)-tartarate into Co(II)-tartarate [22]. Some of the research groups have proposed a weak organic acid-driven spent-LIB leaching mechanism. For example, Li’s group proposed the surface reaction between citric acid, a reducing agent of H_2_O_2_, and LiCoO_2_ particles, and what might be described as a three-tier reaction below [62]. Adding a reducing agent can speed up the reaction.

Tier 1: $6C_{6}H_{8}O_{7}+2LiCoO_{2}+H_{2}O_{2}⇌2{Li}^{+}+6C_{6}H_{7}O_{7}^{-}+2{Co}^{2+}+4H_{2}O+O_{2}$;

Tier 2: $6C_{6}H_{7}O_{7}^{-}+2LiCoO_{2}+H_{2}O_{2}⇌2{Li}^{+}+6C_{6}H_{6}O_{7}^{2-}+2{Co}^{2+}+4H_{2}O+O_{2}$;

Tier 3: $6C_{6}H_{6}O_{7}^{2-}+2LiCoO_{2}+H_{2}O_{2}⇌2{Li}^{+}+6C_{6}H_{5}O_{7}^{3-}+2{Co}^{2+}+4H_{2}O+O_{2}$.

Reaction mechanisms of other organic acid-driven spent-LIB leaching, as shown in Figures S3 and S4, reflected that the organic acid molecules acted as a chelate to form a metal ion complex after dissociate hydrogen ion [20,66,73], which improved the efficiency of the metal leaching from the spent LIBs.

#### 2.2.2. Strong Organic Acids

Aside from the several commonly used mild organic acids mentioned above, there are reports of using mild organic acids, such as iminodiacetic acid, maleic acid, formic acid, succinic acid, aspartic acid, glycolic acid, acetoacetic acid, trichloroacetic acid, acetic acid, benzenesulfonic acid, etc., with reducing agents (H_2_O_2_ or ascorbic acid) for spent-LIB recycling [19,23,26,59,61,63,64,65,74,75,76]. However, some of the previously mentioned organic acids with low acidity (pK_a_ > 0) have the problem of relatively low leaching efficiency compared with mineral acid. The use of alternative organic acids for metal recovery will be the solution. Recently, the use of strong organic acid (pK_a_ < 0) has been shown to be one way to eliminate the adverse effects of secondary pollution arising due to the evolution of toxic gas (e.g., Cl_2_, SO_2_, NO_x_, etc.) and achieve high metal recovery efficiency (~100%). Furthermore, some of the strong organic acids, like methanesulphonic acid (MSA), have been claimed to be biodegradable [61], which can make the process green in nature. The selected candidates for this review are MSA [61], benzenesulphonic acid [59], and p-toluene sulfonic acid (PTSA) [64,65], all of which can achieve high metal recovery efficiency. For example, Fu et al. [59] demonstrated benzenesulfonic acid-assisted leaching in a spent LCO battery. The results showed that the best Li^+^ and Co^2+^ leaching efficiency levels achieved were 99.58% and 96.53%, respectively, at 90 °C, 0.75 M benzenesulfonic acid, and 3 vol% H_2_O_2_ from a 15 g/L LCO black powder/acid mixture. MSA also exhibited strong Li^+^ and Co^2+^ leaching efficiency values of 100% at 70 °C under a high solid-to-liquid ratio (S/L) of 81.2 g/L; the MSA concentration of 2.5 M compared with other weak organic acids (malonic acid, succinic acid, citric acid, and oxalic acid), which carried by Wang’s research team with varying reaction parameters, as illustrated in a corresponding source [61]. Furthermore, the use of an aromatic substituted sulfonic acid was the best way to achieve effective Li and Co recovery from spent LIBs. For example, Liu et al. [64] achieved the best Li^+^ and Co^2+^ recovery efficiency, at levels of ~100% (Li^+^) and ~90% (Co^2+^) under a low solid-to-liquid ratio (S/L) of 30 g/L, and a PTSA concentration of 1.5 M at 80 °C, compared with other weak organic acids, throughout 2 h leaching. All mentioned candidates exhibited spent-LIB leaching activity comparable to the conventional inorganic acid leaching process.

**Table 2:** Activities of organic acid-driven leaching of spent LIBs.

Acid (Concentration (M))	Spent LIBs Target	Reducing Agent (Concentration)	Solid-to-Liquid Ratio (g/L)	Metal Recovery (%)	Ref.
Citric acid (1.25 M)	LCO/NCM	H_2_O_2_ (1% *v*/*v*)	20	Li: 99%, Co: 91%	[62]
Citric acid (1.5 M)	NCM	D-glucose (0.5 g/g)	20	Li: 99%, Ni: 91%, Co: 92%, Mn: 94%	[27]
Citric acid (1.5 M-reused 5 times)	NCM	D-glucose (0.5 g/g)	20	Li: 97%, Ni: 89%, Co: 92%, Mn: 91%	[27]
Ascorbic acid (1.25 M)	LCO	N/A	25	Li: 98.5%, Co: 94.8%	[20]
Lactic acid (1.5 M)	NCM	H_2_O_2_ (0.5% *v*/*v*)	20	Li: 97.7%, Ni: 98.2%, Co: 98.9%, Mn: 98.4%	[9]
Tartaric acid (0.2 M)	LCO	L-ascorbic acid (0.04 M)	N/A	Co: >95%	[23]
Tartaric acid (2 M)	NCM	H_2_O_2_ (4% *v*/*v*)	17	Li: 100%, Ni: 100%, Co: 100%, Mn: 100%	[66]
Tartaric acid (0.2 M)	LCO	L-ascorbic acid (0.04 M)	N/A	Li: 100%, Co: 95%	[22]
Oxalic acid (0.6 M)	NCM	N/A	20	Li: 75%, Ni: 1%, Co: 1%, Mn: 1%	[71]
Oxalic acid (0.3 M)	LFP	N/A	60	Li: 100%, Fe: 8%	[72]
Malic acid (1.5 M)	LCO	H_2_O_2_ (2% *v*/*v*)	20	Li: 99%, Co: 93%	[69]
Malic acid (1.5 M)	LCO	H_2_O_2_ (2% *v*/*v*)	20	Li: 93%, Co: 100%	[63]
Malic acid (1.25 M)	LCO	Glucose (0.3 M)	20	Li: 100%, Co: 99.87%	[70]
Methanesulphonic acid (MSA) (2.5 M)	LCO	H_2_O_2_ (0.9% *v*/*v*)	81.2	Li: 100%, Co: 100%	[61]
Benzenesulphonic acid (0.75 M)	LCO	H_2_O_2_ (3% *v*/*v*)	15	Li: 99.58%, Co: 96.53%	[59]
p-toluene sulfonic acid (PTSA) (1.5 M)	LCO	H_2_O_2_ (0.9% *v*/*v*)	30	Li: ~100%, Co: ~90%	[64]

N/A: No reducing agent.

Besides research involving individual acid candidates, there were comparative studies involving mineral acids and organic acid-driven waste-LIB-powder leaching as part of an industrialization evaluation. For example, spent LCO and NCM333 recycling was carried out by Gao’s team, and Zhang’s team showed that the difference in leaching efficiency (Li^+^, Ni^2+^, Co^2+^ and Mn^2+^) was obvious between mild organic acids (maleic acid, citric acid, succinic acid, ascorbic acid, tartaric acid, DL-malic acid, Im-inodiacetic acid, aspartic acid, and oxalic acid) and mineral acids (hydrochloric acid, sulfuric acid, and nitric acid) under the same leaching conditions (acid concentration, solid-to-liquid ratio (S/L), temperature, and presence of reducing agents (e.g., H_2_O_2_)). The chance of Al being recovered into the acid from the foil residue will be very high and the leaching process will be faster when inorganic acids are used, due to their higher acidity relative to organic acids [75,77]. The reason behind this may be reflected in the nature of the leaching process, as shown in the illustration in [77]. Another example is the leaching activity shown towards NCM523 and a composite materials battery (CMB: 70% NCM523 + 30% LMO dopant) by a strong acid (2 M phosphoric acid) and organic acids (1 M citric acid and 1 M acetic acid), as demonstrated by Song’s team [37]. The results showed that a strong mineral acid (phosphoric acid) had a higher leaching efficiency towards NCM523 (Li: 95.4%, Ni: 99.8%, Co: 99.5%, Mn: 98.0%) and CMB (Li: 92.9%, Ni: 93.3%, Co: 94.8%, Mn: 94.3%) than shown by citric acid (NCM523: Li: 91.0%, Ni: 85.7%, Co: 99.9%, Mn: 93.6% CMB: Li: 88.2%, Ni: 72.5%, Co: 94.0%, Mn: 90.5%) under identical optimized leaching parameters (reducing agent: 5 *v*/*v*% H_2_O_2_, solid-to-liquid ratio (S/L): 25 g/L, temperature: 40 °C, time: 60 min). Acetic acid was the worst among the three candidates. The reaction mechanism of the leaching process was also different due to the acid anion involved; the only common point was that the metal ions were being chelated by the acid anionic center, as proposed by Song’s group; please refer to the original article’s text for details of the products formed between the metal ions and the anions from the acid candidates used [37].

By integrating the comparative study results in Table 1 and Table 2, it can be seen that even though mineral acid has better performance, even without a reducing agent, compared to cases driven by organic acids, secondary pollution due to discharge of residual chemicals (reducing agent, acidic waste water) and the instability of the reducing agent were still the problem. This comes from the relatively higher concentration of acid that is required to drive the acid leaching with mineral acid (2–4 M), compared to the mild organic acid-driven reaction systems (1–2 M). Li et al. [78] even need to use 6 M of hydrochloric acid for the iron stripping step before the H_2_SO_4_ (3 M)-driven spent-NiMH leaching. In contrast, organic acids result in a relatively mild residual solution, while overall leaching efficiency is strongly reduced even with the help of reducing agents, while the two together increase the cost. Such results show the double-edged-sword properties of hydrometallurgy driven by organic acids and mineral acid, and indicate the importance of choosing correct acid and reducing agent combinations during the upgrade to industrial-scale operations.

Furthermore, relatively few efforts were available addressing the reusability of acids in spent-LIB recycling [27], as the acids used in most of the cited examples were used only once (Table 1 and Table 2), which may violate the green and sustainability principles of a continuous spent-LIB recycling production line to be constructed in the future. The need for more extensive study on acid (leachant) recycling should be emphasized.

### 2.3. Kinetics of Leaching

Leaching kinetics have played an important role in identifying rate-controlling steps in metal dissolution with low leachant consumption and low emissions, and the characterization of the kinetics of acid leaching of valuable metals in spent LIBs has gained attention in recent years [1,59,79]. Scholars are turning their attention to the investigation at the mechanistic level, including dynamics [37]. In general, leaching of metal in acid solution is a solid–liquid heterogeneous reaction that occurs on the outer surface of the unreacted particle [59]. The leaching rate may be controlled by diffusion through a fluid film, the ash or product layer, or a surface chemical reaction [75]. The classical shrinking core model (SCM) is usually employed to describe the leaching kinetics of Co and Li from spent LCO [80]. If the leaching process is controlled by the chemical reaction [81], the SCM can be expressed as Equation (1):

(1) $${\left(1-f\right)}^{\frac{1}{3}}=1-kt$$

And if the leaching process is diffusion-controlled in the SCM, this can be expressed as Equation (2) with example plots in the organic acid-assisted NCM333 leaching, as shown in reference [82]:

(2) $$3{\left(1-f\right)}^{\frac{2}{3}}-2(1-f)=1-kt$$

where *f* is the leaching efficiency of a metal, *k* is the reaction rate constant (min^−1^), and *t* is the leaching time (min).

According to the references, about 40 kinetics models have been reported; these can be grouped into five major types: diffusion, reaction-order, nucleation, geometrical contraction, and power function [83,84]. Aside from the most commonly used SCM, the other two kinetic models of metal ion leaching process, the Avrami equation and logarithmic rate law, are frequently used [75,80]. When the fitting degrees for Li, N, Co and Mn (R^2^ values) are relatively low (<90%) according to the fitting results, other kinetic models can be used for fitting, and an optimal R^2^ value is selected as the model of the metal leaching kinetics. The Avrami equation expressed in Equation (3) was originally developed and validated for crystallization kinetics in solution, and crystallization can be regarded as the reverse process of leaching [75], allowing the equation to be used for the description of leaching kinetics.

(3) $$ln(1-x)=-kt^{n}$$

where *x* is the fraction reacted, *k* is the specific rate constant (min^−1^), *n* is the suitable parameter and *t* is the reaction time (min).

From the leaching point of view, due to the oxidation–reduction phenomenon during the leaching reactions (LCO and H_2_O_2_ as oxidant and reductant, respectively), a logarithmic rate law model can be more appropriate [80]. The logarithmic rate law model can be expressed as Equation (4):

(4) ln(1−x)=−kt

where *t* is leaching time and *x* is the fraction, while *k* is the logarithmic rate constant.

Huang et al. [85] used four different kinetic models for fitting and comparison, and found that the leaching kinetic curves of Li, Ni, Co and Mn were basically the same, while the Avrami equation model showed the best correlation fitting for the leaching process. The article by Chabhadiya et al. [86] found that although the SCM model is a suitable model for describing leaching kinetics, when the data were evaluated to fit with different kinetic models for the solid–liquid mass transfer reaction, the R^2^ value of the logarithmic rate law model was higher, according to kinetics plots in the original text of the article. This indicated that the logarithmic rate law is a more suitable metal leaching model [86]. Therefore, choosing the most appropriate model depends on experimental observations rather than the hypothetical model.

The relation between reaction rate constant and the temperature can be given by the empirical Arrhenius law (Equation (5)), and through the Arrhenius equation, the activation energy (E_a_) can be determined [59,75].

(5) $$K=Ae^{\frac{{-E}_{a}}{RT}}$$

where *K* (min^−1^) is a reaction rate constant, *A* is the pre-exponential factor, *E_a_* (kJ/mol) is the apparent activation energy, *R* is the universal gas constant and *T* (K) is the leaching temperature.

Usually, the activation energy of the leaching reaction can be estimated by expressing the Arrhenius equation in linear form [59,75,81,85]. According to the results summarized in Table 3, activation energy refers to the minimum extra energy required for the compounds to result in a chemical reaction. Therefore, the smaller the value of *E_a_*, the faster the reaction rate and the quicker it is to react [85]. For example, the E_a_ values for the leaching of Li, Ni, Co and Mn were determined as 28.00, 44.51, 44.79, and 43.81 kJ/mol, respectively. This means that Li is being leached most easily, followed by Mn, Ni and Co [75]. Chabhadiya et al. [86] mentioned that the *E_a_* value of Cu is significantly higher than those of Li, Ni, Co and Mn, which indicated that copper initially forms insoluble products and needs more energy to overcome the energy barrier. When the *E_a_* of metal ions is greater than 40 kJ/mol, the rate-controlling step during the leaching process belongs to the surface chemical reactions for the metal ions [85]. He et al. [66,81] found that the *E_a_* values for the leaching of Mn, Li, Co, and Ni were 66.00, 54.03, 58.18, and 73.28 kJ/mol in the first stage, respectively. Such results revealed that the dissolution of cathode metals was controlled by the surface chemical reactions. In contrast, Chabhadiya et al. [86] mentioned that the determined *E_a_* values for the leaching of Ni, Co, Mn and Li are 11.2, 9.5, 6.2 and 9.7 kJ/mol, based on the kinetic plots in the original work, respectively. Such results indicate that the dissolution of cathode metals was diffusion-controlled [86].

## 3. Separation and Recovery

The separation and recovery methods for metal elements in solutions usually include selective extraction, chemical precipitation, electrodeposition, salting out and ion exchange.Extraction or chemical precipitation methods are usually used to enrich and separate Li, Ni, Co and Mn ions from wastebatteries. Extraction or chemical precipitation methods are usually used to enrich and separate metal ions. Chemical precipitation refers to the formation of insoluble compounds from valuable metal ions in the leachate with different precipitants, due to differences in the solubility levels of valuable metal ions, followed by separation and purification. The co-precipitation method can process a large amount of waste metal ions. The separation of metals can be achieved by controlling the pH value. Such processes are easy to realize within industrialization. However, it is easily interfered with by ions from metal impurities, which results in lower product purity compared with the extraction method. Therefore, the key to this process is to select a precipitant with better selectivity and further optimize the process conditions to control the sequence of precipitation of valuable metal ions, thereby improving the purity of the product.

The selective extraction of spent-LIB cathode materials is usually in a liquid–liquid extraction system; this refers to the use of extractants to extract the required elements from the leachate, and the specific metal ions are extracted from the aqueous phase to the organic phase by using the differences in solubility or distribution coefficients of metal ions such as lithium, nickel, cobalt, and manganese in the organic phase and the aqueous phase. Common extractants include PC-88A, Cyanex 272, D_2_EHPA, P507, Acorga M5640, Aliquat-336, TBP, and so on; the corresponding chemical structures of selected solvents are illustrated in Figure S6 [34]. Among these, Cyanex 272 is the most commonly used. Comparisons of the extraction rates of PC-88A, Cyanex 272, and D_2_EHPA found that in the extraction results from weakly acidic sulfate solutions the separation factor between Ni and Co increases in the following order: D_2_EHPA < PC88A < Cyanex 272 [88]. Studies on the extraction of Co from chloride and sulfate media include those using a single extractant such as Cyanex 272 [32,89], D_2_EHPA [90,91], or PC-88A [92], or mixtures of extractants, such as Cyanex 272 and PC-88A [52] or D_2_EHPA and LIX 63 [88]. The solvent extraction method has the advantages of low energy consumption and good separation effect. The acid leaching–solvent extraction method is currently the mainstream process for treating spent LIBs in industry. However, the selection of extractant and the further optimization of extraction conditions still constitute the current research focus in this field, in order to achieve more efficient, environmentally friendly and recyclable treatments. In addition to the use of chemical precipitation or extraction alone for the extraction of valuable metals, sometimes the two methods are combined for extraction together. For example, in the article of Rao et al. [93], first, the chemical precipitation method was used to reduce the contents of Al^3+^ and Fe^3+^, and then the contents of Al^3+^, Fe^3+^ and Mn^2+^ were further reduced by P204 extraction. Finally, P507 was used for tertiary extraction to separate the Ni^2+^ and Co^2+^. Another example is metal recovery from the spent NCM black powder, which involved the use of D_2_EHPA and Cyanex 272 for separation of Mn^2+^ (D_2_EHPA) and Ni^2+^, Ni^2+^ and Li^+^ from the acid leachate in different stages, as proposed by Tang’s research team, and shown in Figure S7 [94].

### 3.1. Extractant

#### 3.1.1. PC-88A and P507

PC-88A (2-ethylhexyl phosphonic acid-mono-2-ethylhexyl ester) was first developed by the Daihachi Chemical Industry and sold by Shell. PC-88A is an excellent extractant for solvent extraction of various metals, comparable to D_2_EHPA in the separation of Co and Ni under different pH values. The acidic forms of the extractants D_2_EHPA and PC-88A have low extraction efficiencies for Mn(II) and Co(II) ions, which led to the use of the sodium form of the extractants [95]. Generally, NaOH and PC-88A (Na-PC88A) are usually used for extraction in kerosene solution. The extraction rates of Zn, Co, Mn and Ni increased with the increase in the saponification rate of the extractant, and the extraction order was Zn > Mn > Co > Ni [95]. In recent years, many Chinese scholars have conducted a lot of detailed research on PC-88A. For example, Liu et al. [88] studied the effects of initial pH value, extractant concentration and saponification degree on metal extraction and separation, and found that Co was preferentially extracted over Mg and Ni by saponified PC-88A and Cyanex 272. When the reaction temperature of the extraction system is 40 °C, the oil–water ratio (O/A) = 1/1, the concentration of the extractant 80%, the initial pH = 6.5, and the saponification rate of the extraction agent PC-88A is 10%, the extraction efficiency of Ni can reach 98.0% [96]. In their research, Nguyen et al. [97] determined that the most optimal conditions for the extraction of Co^2+^ were when the pH value is close to 4.5, the O/A ratio is 3:1, the stirring time is 10 min, the stirring speed is 600 r/min, and the temperature is 24 °C, and the extraction rate is as high as 99.8%.

**Figure 3:**
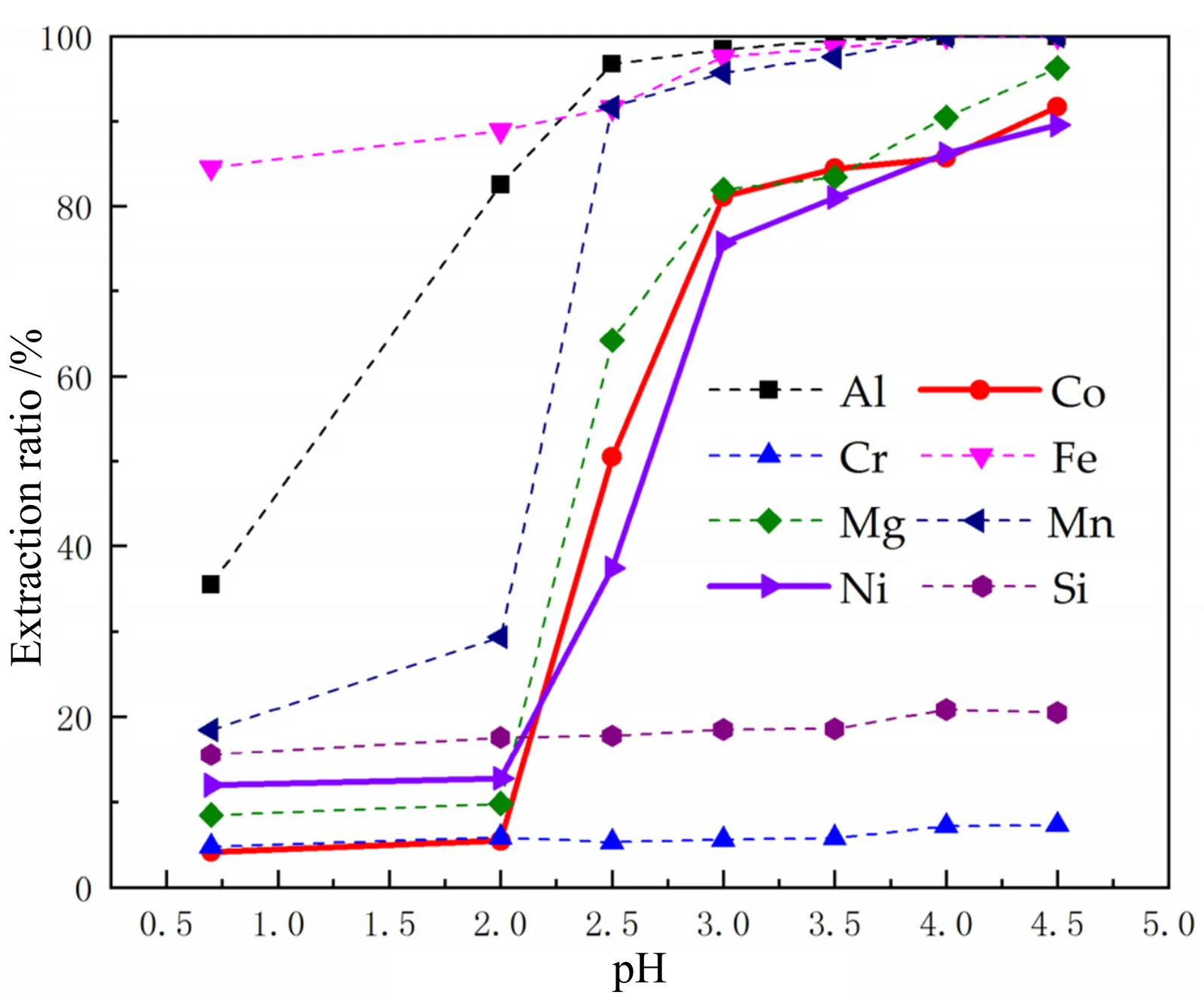
Effect of pH on the extraction of metals with P507 [93]. (Reproduced with permission, 2020, MDPI).

P507 (2-ethylhexyl phosphonic acid mono-2-ethylhexyl ester) was produced in the early 1970s, and in 1981, a commercial application of Co/Ni separation was realized in a Chinese metallurgical enterprise [92]. There are still few studies on the application of P507 extractant in waste batteries to date. The separation of Ni and Co can also be achieved by using P507 extractant. For example, the selective extraction of Ni^2+^ and Co^2+^ can be realized by using the P507 extractant, as demonstrated by Rao et al. [93] with achieved extraction rate of Co^2+^ and Ni^2+^ of 96.61% and 12.32%, respectively. Since P507 is an acidic extractant, the saponification rate of P507 has little effect on the extraction rates of zinc and nickel, while the extraction rates of copper and cobalt increase with the increase in saponification rate [98]. Wu et al. [98] used sodium-saponified P507 extractant to separate copper, zinc, and cobalt. The extraction rates of copper, zinc, and cobalt were 96.73%, 99.87% and 94.17%, respectively, with a saponification rate of sodium of 65%, O/A ratio of 1:1, and an equilibrium pH of 4. The pH dependence towards Ni^2+^ and Co^2+^ extraction performance in Rao’s demonstration is illustrated in Figure 3, which showed that the best metal extraction (Al: 98.53%, Mn: 97.87%, Co: 92% against Fe: 87.71%, Mg: 77.75%, Ni: 57.71%) was achieved at pH 3 by P507 [93].

#### 3.1.2. Cyanex 272

Cyanex 272 ((bis-2, 4, 4-trimethylpentyl) phosphinic acid) was developed by Cytec Chemicals [92], and is the most commonly used extraction agent in separating the valuable metals Ni, Co and Mn from waste lithium-ion batteries. As a result, there are many published research efforts using and performing research on Cyanex 272. In most cases, cobalt and nickel in sulfate media were separated by saponified Cyanex 272. Swain et al. [89] have done a lot of research on the Cyanex 272 extractant from the aspects of pH and the concentration of Cyanex 272, and they found that lithium recovery was very low compared to cobalt extraction. The results show that the extraction rate of Co is greatly improved with an increase in the concentration of Cyanex 272, while the extraction rate of Li is only slightly increased [89]. The O/A volume ratio of Cyanex 272 also influences the extraction rates of Co and Li to a certain extent. As the O/A ratio increases from 1.0 to 2.0, the extraction rate of Co increases from 60% to nearly 100%, and the extraction rate of Li increases from 10% to about 40%. The extraction process begins by making an optimum emulsion phase (10% *w*/*v* SPAN 80 and 0.7 M Cyanex 272) and for extraction, the best extraction efficiency is achieved when the feed phase pH is 5 for Co and 6 for Mn, with the highest extraction efficiency values for Co and Mn being 88.54% and 89.28%, respectively [56]. Ahmadipour et al. [99] wrote that when using 20% Cyanex 272 at 25 °C and a pH of 5, the extraction efficiency of Mn can reach 100%. In addition to their recycling value in LIB cathodes, recovered cobalt species have broad relevance to sustainable energy technologies. For instance, atomically dispersed CoN_x_ active sites synergizing with Co nanoparticles have been shown to greatly enhance hydrogen generation from ammonia borane hydrolysis [6]. Such examples underscore the strategic importance of cobalt recovery, directly linking battery recycling to future hydrogen economy applications.

Since there are different metal elements in the solution (e.g., Cu^2+^, Fe^2+^, Al^3+^, etc.), different extractants are usually used together in order to ensure the sole extraction of Co. For example, in the article by Nan et al. [100], Acorga M5640 was used to extract 97% of Cu as a first step, 10 wt% NaOH was then used to remove Fe^3+^ and Al^3+^ from impurities by forming precipitates of Fe(OH)_3_ and Al(OH)_3_ as a second step, and 1 M Cyanex 272 was selected as the extraction agent for extracting Co in the final step, after the impurities were removed. In the study by Kang et al. [101], copper, iron and aluminum were firstly precipitated from the solution in the form of hydroxides by adjusting the pH value to 6.5; this was followed by selective cobalt extraction from the purified aqueous phase by equilibrating with 50% saponified 0.4 M Cyanex 272 at an equilibrium pH of 6, whereupon 92% of the cobalt could be recovered from the spent LIBs.

#### 3.1.3. D_2_EHPA

D_2_EHPA (di-2-ethylhexyl phosphoric acid) was the first commercial process, developed by Ritcey et al. [102]; organophosphorus extractants have been shown to be the main solvents for the separation of cobalt and nickel in acidic media solutions. Since D_2_EHPA extractant has the advantages of high efficiency and low cost, D_2_EHPA has been used on a commercial scale, mainly as a method for recovering zinc from secondary materials [99,103,104,105]. When D_2_EHPA was at a low pH (below 2.5), D_2_EHPA preferentially extracted zinc, but the extraction rates of both zinc and manganese increase as the pH increases [99,104]. In a study by Vahidi et al. [104], it was found that zinc extraction increased from 10% to ca. 99% when D_2_EHPA concentration increased from 5 to 40% (*w*/*w*), while the A/O ratio may also affect the extraction rate. When the pH value was below 2.5, the A/O changed from 4/1 to 1/1, which increased the percentage of zinc extraction. A study by Wang’s group found that the extraction rate of zinc was relatively low when unsaponified, and the extraction rate of nickel and zinc gradually increased with the continuous increase in saponification rate [106]. When the equilibrium pH is 2.76 and the saponification rate is 59%, the extraction rate of zinc is close to 100% [106].

Due to the low selectivity of D_2_EHPA for the separation of Zn and Mn at low pH, it was necessary to use a secondary extractant as a synergist [103,107]. Hooseini et al. [103] used experimental data from D_2_EHPA and Cyanex 302 as co-extractants to extract zinc and manganese experiments to establish a zinc and manganese separation model. Ahmadipour et al. [99] found that the mixed extraction of D_2_EHPA and Cyanex 272 has a synergistic extraction and separation effect on zinc and manganese and mixing the two extractants can improve the extraction capacity of the mixture; 1/3 *v*/*v* D_2_EHPA/Cyanex 272 mixture exhibited the best Mn and Zn separation. In addition, D_2_EHPA can not only extract and separate zinc and manganese; Zhang et al. [108] confirmed that isooctyl alcohol as a synergistic extractant has an obvious synergistic effect on the extraction of chromium, and used an extractant composed of D_2_EHPA and isooctyl alcohol to extract and recover chromium (III). With increases in D_2_EHPA concentration and initial pH, the extraction of chromium reached its maximum at a higher iso-octanol concentration [108]. The mixed extractant of D_2_EHPA and LIX63 can effectively extract and separate Ni(II) and Co(II) from the chloride leaching solution of nickel laterite ore containing Al and other impurities, and Ni is preferentially extracted in high-concentration HCl or H_2_SO_4_, while Co is preferentially extracted in low-concentration acid [88].

### 3.2. Purification

#### 3.2.1. Solution-Based Purification

The presence of impurities (e.g., Al, Cu, and Fe) in hydrometallurgy products will affect the performance of regenerated LIB cathodic materials [109]; the impurities must be removed from the leachate, so the purification step plays an important role. One of the extensively studied and implemented purification techniques is a solvent-based method via solvent extraction which operates by means of the ion separation reaction with the solvent. The list of widely used solvents for removing impurities includes Acorga M5640 [100] and P507 [93], respectively. For example, multivalent ion (Al^3+^, Fe^2+^, and Cu^2+^) impurities can be effectively separated from the NCM leachate under careful control of pH (pH 2), as shown in Rao’s demonstration results shown in Figure 3 [93], where >80% Al^3+^ and Fe^2+^ were extracted from the mixture by P507 and NCM ions still in the leachate (extraction ratio < 30%). Such results can be regarded as the reference case of battery impurity elimination throughout hydrometallurgy.

#### 3.2.2. Purification via Membrane-Based Selective Separation of Metal Ions

Since the use of conventional purification technology suffers from weaknesses, including environmental threats, long operation times, and poor recovery efficiency [110], especially in dealing with purifying multivalent metal ions (Ni, Co, Mn) in leachate containing monovalent Li^+^ throughout hydrometallurgy. The use of membrane purification becomes one of solutions due to its easy operation, space- and chemical-saving aspects, and high treatment efficiency [111], with a reference scheme having been proposed by Yao et al. [112], as illustrated in Figure S8. To date, nanofiltration (NF) is the commonly used technology due to the strong selectivity of these approaches towards different metal ions and effective separation according to the ion size between Li^+^ and NCM ions in the mixture, in addition to the ion exchange reactions between metal ions in the leachate and the cationic exchange functional groups located on the membrane surface, with pore size (diameters) in nm dimensions [112,113,114,115]. Demonstration from different groups showed that chemically modified commercial NF membranes (NF1000-EDA [112], VNF-4040 [114], and PA-M_2_ [113]) and some as-used commercial membranes (Desal-DL, Desal-KH) [115] exhibited strong separation ability for Li^+^ with respect to the rest of the multivalent metal ions in various types of spent-LIB leachate in acidic pH (1–2), with recorded rejection percentages in the range of 1.5–64.8% for Li vs 47–99.9% for multivalent ions or a separation factor in the range of 2.11–4.03 (overall view) to 307.4, and to 645.9 (individual ion-based) [112,113,114,115], as summarized in Table 4. Such characteristics were reflected in the Li^+^ and NCM metal separation performance, and the surface functional group configuration illustrated in Figure S9 is representative [112]. The most important criterion for the membrane used is acid resistance, due to the fact that spent-LIB leachate generally is highly acidic (pH < 2) [110,112,113,114,115]. However, such methods suffer from a weakness in that the actual impurities in the leachate (e.g., Fe^2+^, Al^3+^, and Cu^2+^) cannot be filtered by the membrane due to the fact that these are also multivalent species and have particle sizes similar to those of NCM, and therefore will also be extracted with NCM ions by the ion exchange mechanism. Such a phenomenon is partially reflected in the low rejection percentage difference between NCM metal ions and Fe^2+^, Cu^2+^, and Al^3+^ (impurities) in four commercial NF membranes throughout the simulated NCM leachate filtration (HCl, as an example) by alkaline reagents of NaOH and Mg(OH)_2_, as shown in Table 4, and the descending order of rejection percentage is (Al, Fe > Mg, Mn, Co, Ni, Cu > Li) [115]. As a result, NF of spent-LIB leachate can only be regarded as an enhancement of the solvent-driven purification method [110] instead of an impurities-removal process.

In summary, it is important to achieve a balance as to the use of the extractant and filter-based methods to purify impurities in the spent-LIB leachate, with the largest goal being to produce high-quality regenerated NCM final products. This is especially important in the industrial-scale hydrometallurgy operation, in order to emphasize green metal recycling by minimizing wastage of chemicals (extractants) and the leaking of valuable metals from the leachate throughout the spent-LIB recycling process.

### 3.3. Chemical Precipitation Method

**Figure 4:**
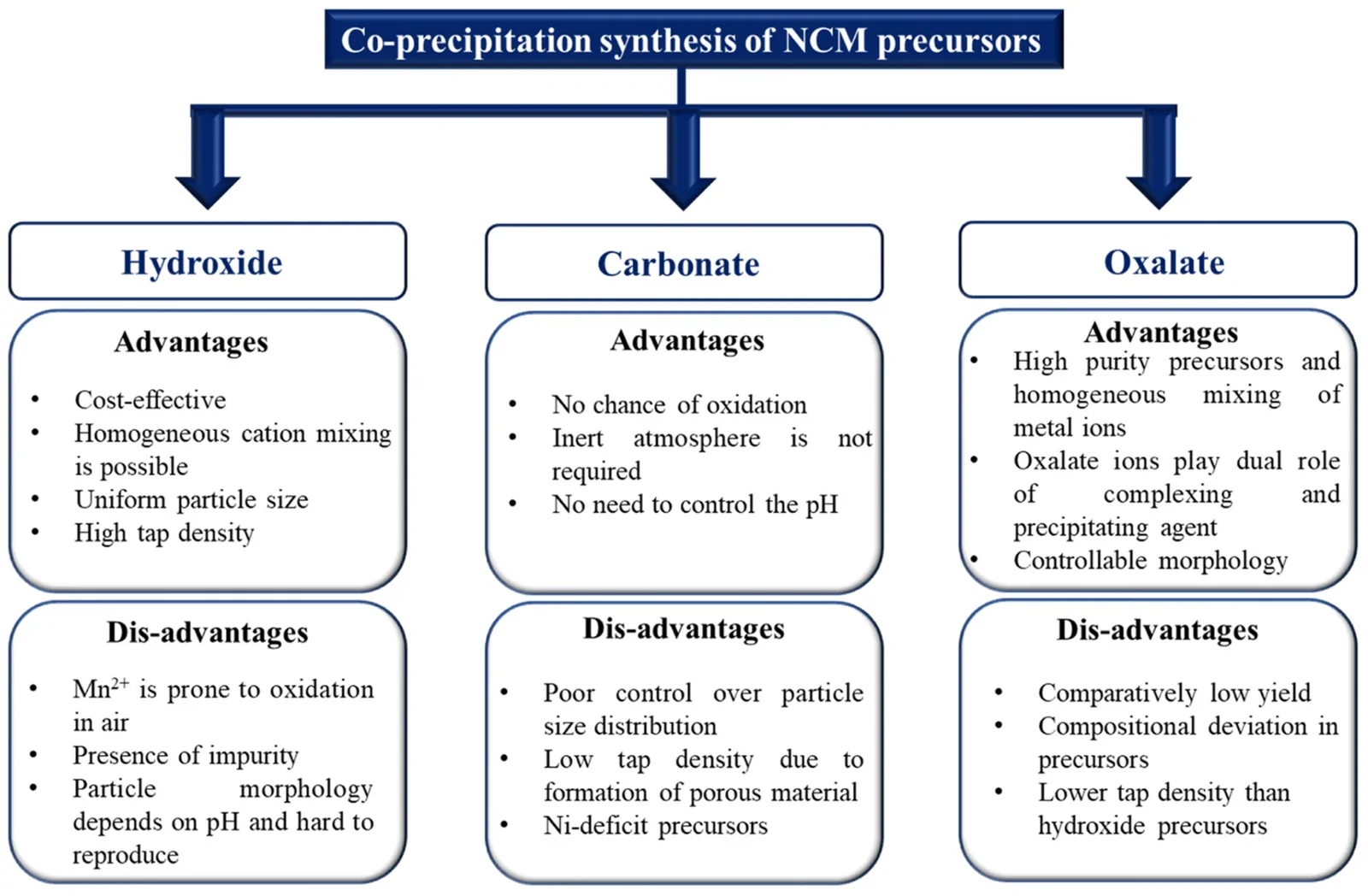
Advantages and disadvantages of various co-precipitation methods used to produce NCM precursors [128]. (Reproduced with permission, 2023, RSC publication).

The chemical precipitation method has the advantages of convenient operation and simple process, and it is considered as the most facile, efficient, and inexpensive technique. However, a large amount of waste residue will be generated in the process of heavy metal treatment, which is very likely to cause secondary pollution if the products are not treated twice. Currently, chemical precipitation recovery is an important method for industrialized recovery of ternary system waste batteries [116]. The chemical precipitation method normally refers to the co-precipitation method, which has a wider range of applications. The co-precipitation method is mainly used to adjust the original solution containing nickel, cobalt, manganese and other elements to a suitable ratio and then co-precipitate them. The oxides or salts (Li, Co, Ni, etc.) are recovered through precipitation and then recycled into chemical raw materials. Co-precipitation processes require tight control of several parameters, such as pH, temperature, and complexing agents, to obtain the desired material [116]. It is well known that the layered LiNi_x_Co_y_Mn_z_O_2_ can be synthesized in various ways, such as hydroxide co-precipitation [65,117,118], carbonate co-precipitation [119,120,121,122,123], and oxalate co-precipitation [124,125,126,127], by using co-precipitation agents, such as sodium hydroxide, sodium carbonate, and oxalic acid. As a result, choosing the best co-precipitation for the regeneration of LIB cathodic materials based on the characteristics of each co-precipitation method, as summarized in Figure 4, is the key to success [128].

#### 3.3.1. Hydroxide Co-Precipitation Route

**Figure 5:**
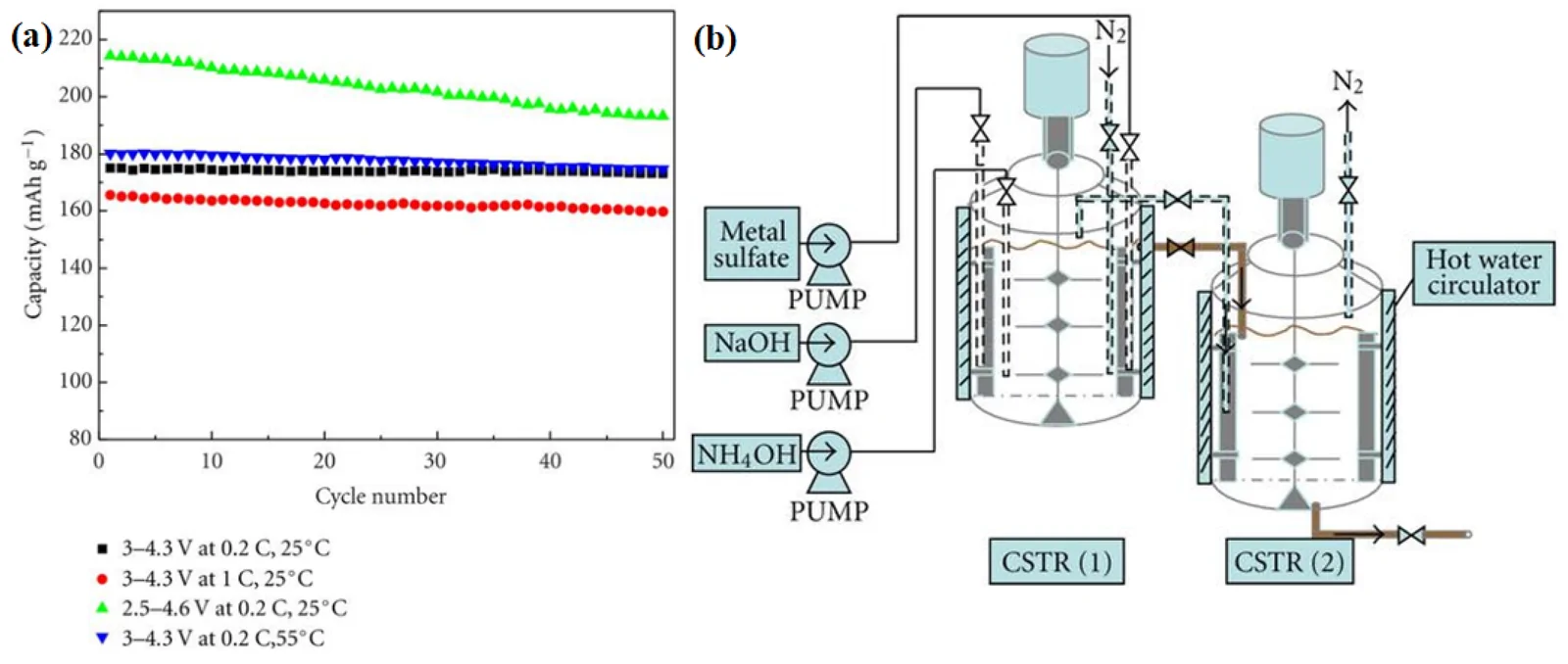
(**a**) Specific discharge capacity of Li/Li[Ni_0.5_Mn_0.3_Co_0.2_]O_2_ cell as a function of cycle number under different charge/discharge conditions. (**b**) Schematic of device for preparing [Ni_0.5_Mn_0.3_Co_0.2_](OH)_2_ precursor by continuous coprecipitation [118]. (Reproduced with permission, 2012, Hindawi Publishing Corporation).

The hydroxide co-precipitation method is mostly adopted to make a homogeneous precursor and is commonly employed for the preparation of new materials via a liquid–solution transition [117,126]. However, some precipitated transition metal hydroxides are easily oxidized in aqueous solution [117]. For example, the resulting Ni_1/3_Co_1/3_Mn_1/3_(OH)_2_ precursor is unstable; Mn(OH)_2_ is easily oxidized to MnOOH and/or MnO_2_ during co-precipitation, thereby reducing the uniformity of the precursor [117,120,121]. Therefore, the question of how to control the valence state of Mn in aqueous solution is crucial in obtaining uniform LiNi_x_Co_y_Mn_z_O_2_ materials. In an article by Hu et al. [117], it is mentioned that co-precipitation should take place in a mixed solution containing both transition metal ions and NH_3_·H_2_O solution, because the ligand NH_3_·H_2_O can significantly reduce the concentration of free ions Ni^2+^, Co^2+^ and Mn^2+^ due to formation of complex groups, and promote their co-precipitation, thereby forming a homogeneous (Ni_1/3_Co_1/3_Mn_1/3_)(OH)_2_ phase. One of the proposed reaction schemes for hydroxide co-precipitation is illustrated in Figure S10 [129]. However, ammonia is easily volatilized into the air when high temperature (usually 50 °C) and high stirring speed are implemented [130]. Therefore, the hydroxide co-precipitation process should be carried out in an inert atmosphere to prevent the formation of inhomogeneous precipitates with various crystal structures and compositions [116,130]. In addition, controlling the pH value is also important for co-precipitation. For example, the co-precipitated solution was kept at 50 °C for 24 h with pH controlled at 10–11 in Santhanam’s demonstration [124]. In the demonstration by Yang et al. [118], in order to obtain ideal NCM battery materials with high initial discharge capacity (175 mAh g^−1^, 0.2C), excellent cycle performance (98.8% capacity retention after 50 cycles of operation) as shown in Figure 5a, and high volumetric energy density, they prepared spherical Li[Ni_0.5_Mn_0.3_Co_0.2_]O_2_ and used two similarly structured continuous stirred tank reactors (CSTR), as shown in Figure 5b, to control the availability of the precursor morphology.

Recently, due to emphasis on green processes (room temperature, ambient environment), the tailorable NCM ratio has become an important key for spent NCM LIB recycling in green way. Tsang et al. [65] demonstrated hydroxide co-precipitation-based tailorable NCM black powder regeneration with spent commercial LIB black powder mixture (NCM and LMO) under room temperature and pressure. The tailorable NCM (333, 523, 532, and 622) was synthesized by adjusting the desired amounts of Ni^2+^, Co^2+^, and Mn^2+^ salts in the PTSA-assisted LIB black powder leachate with help of ICP-OES monitoring, followed by direct addition of the desired amount of NaOH pellet to the solution adjusted for pH and NCM ratio to achieve NCM hydroxide precursor (Ni_x_Co_y_Mn_z_(OH)_2_) formation. The final NCM products, with a different NCM ratio, were obtained by Li_2_CO_3_ calcination (900 °C) and showed comparable battery (coin cell) performance in 50 cycles cyclic charging/discharging operation (0.1 C) with the best candidate being R622 (196.6 mAh g^−1^) as illustrated in Figure S11 [65].

#### 3.3.2. Carbonate Co-Precipitation Route

It is well known that the Ni_x_Co_y_Mn_z_(OH)_2_ precursor is unstable when using hydroxide co-precipitation. In contrast, the valence state of the Mn ion is always 2+ throughout the carbonate co-precipitation method, which is stable in aqueous solution and improves reproducibility [120,121,122,123]. In addition, the carbonate co-precipitation method is less costly than the hydroxide co-precipitation method. He et al. [121] obtained (Ni_1/3_Co_1/3_Mn_1/3_)_3_O_4_ by adding regenerated NCM leaching solution (1.8 M), Na_2_CO_3_ aqueous solution (1.8 M) and a specific amount of NH_3_·H_2_O into a CSTR simultaneously with stirring speed at 700 rpm under the conditions of pH 7.5 and temperature 60 °C for 12 h, and calcined at 500 °C in air for 5 h. During the carbonate co-precipitation process, the recoveries of Ni, Co and Mn achieved were 97.78%, 99.16% and 99.12%, respectively [121]. A battery fabricated with regenerated NCM111 via carbonate co-precipitation exhibited excellent initial discharging capacity (135.1 mAh g^−1^) with excellent capacity retention (94.1%) after 50 cycles of operation [121]. In order to obtain (Ni_1/3_Co_1/3_Mn_1/3_)CO_3_ carbonate precursor, Park et al. [122] pumped 2 M aqueous solutions of NiSO_4_, CoSO_4_ and MnSO_4_ (Ni:Mn:Co = 1:1:1 molar ratio); 2.0 M Na_2_CO_3_; and 0.2 M NH_4_OH solution into the CSTR reactor at the same time, stirred at a constant speed of 1000 rpm and kept the solution pH at 7.5. The demonstration by Zhang et al. [123] differed from others in that NCM leaching solution, NaCO_3_ solution and NH_4_OH solution were added to a reactor which was filled with CO_2_ gas. Cho et al. [120] successfully calcined Ni_1/3_Co_1/3_Mn_1/3_CO_3_ precursor at different temperatures (750–950 °C) and synthesized the layered Li[Ni_1/3_Mn_1/3_Co_1/3_]O_2_ cathode with spherical-particle-shaped materials. Homogeneous materials can be synthesized even at a low temperature of 750 °C, but the optimal calcination temperature was found to be 800 °C throughout the battery performance test [120].

**Figure 6:**
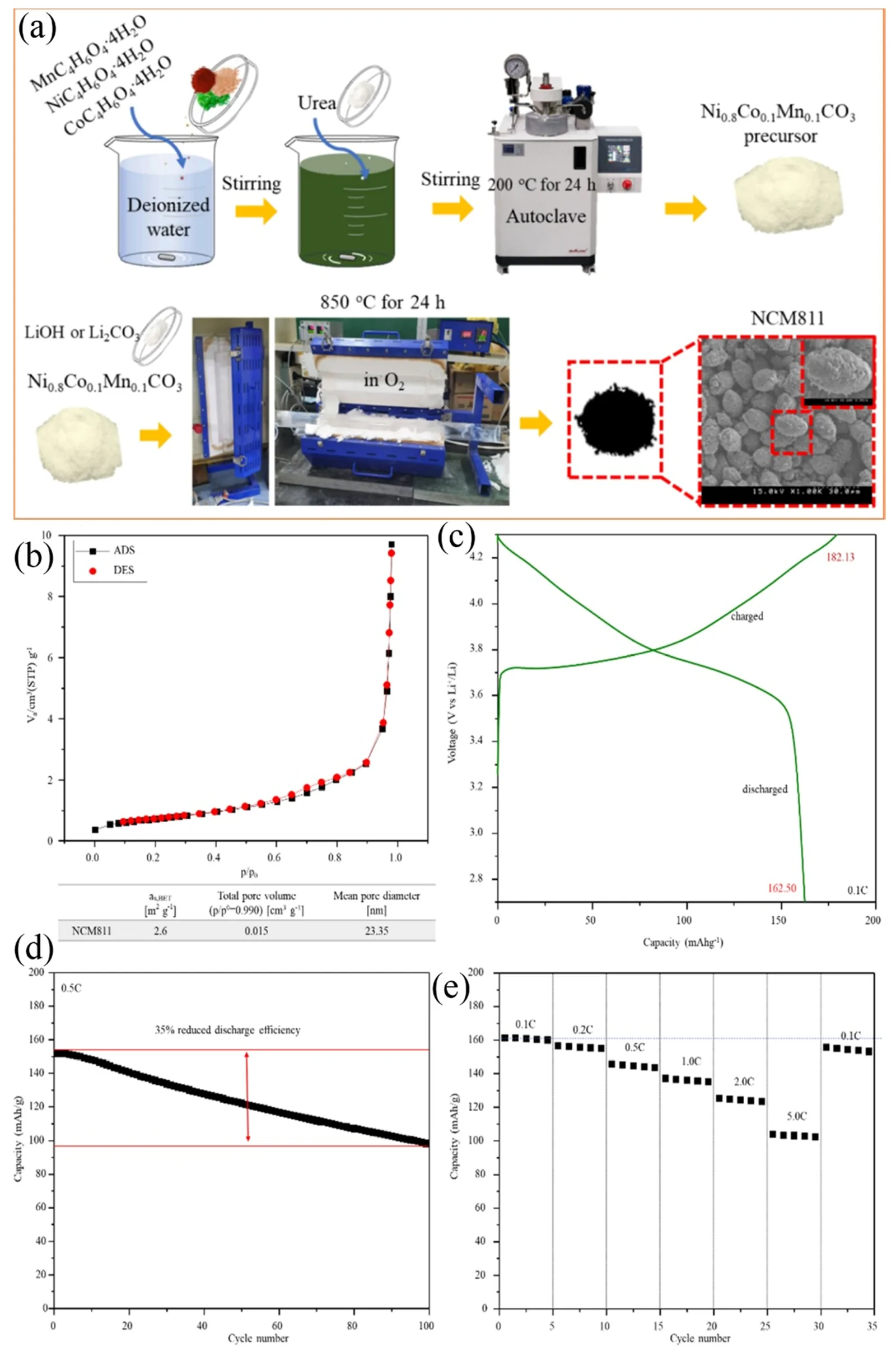
(**a**) Schematic of the synthesizing process of NCM811. (**b**) Isothermal nitrogen desorption/adsorption curve; (**c**) charge/discharge curve acquired at 0.1 C; (**d**) cycle evaluation conducted at 0.5 C; and (**e**) recovery evaluation performed by changing the C-rate for the NCM811 sample prepared under the following optimal conditions: ratio of metal (M):urea = 1:2, use of Li_2_CO_3_ as the lithium source, calcination temperature of 850 °C, calcination time of 25 h, and ratio of NCM:Li = 1:1.05 (mol/mol) [131]. (Reproduced with permission, 2022, MPDI.).

In addition to using Na_2_CO_3_ as co-precipitating agent, lithium-based sources themselves, including Li_2_CO_3_, were also used as co-precipitating agents. For example, synthesis of nickel-rich NCM cathodic materials (NCM 811) by Park’s team with the reaction scheme shown in Figure 6a was carried out by adding Li_2_CO_3_ to the optimized urea-buffered NCM precursors (Ni_2_C_4_H_6_O_4_·4H_2_O, Co_2_C_4_H_6_O_4_·4H_2_O, Mn_2_C_4_H_6_O_4_·4H_2_O vs. urea = 1:2 mol/mol) after mixing. Subsequent autoclave heating formed NCM811 under high temperature calcination in oxygen (800 °C) [131]. The resulting NCM811 (black powder in red square of Figure 6a) exhibited strong performance in the battery test (initial charging/discharging capacity (0.5 C): 182.13 mAh g^−1^/162.50 mAh g^−1^; capacity reduction: 35% after 100 cycles (0.1 C) (red line boundary of Figure 6d); strong capacity recovery rate at 35 cycles C-rate test: 95.56% (blue line of Figure 6e)), and benefited from its excellent BET (2.6 m^2^ g^−1^) and porosity (pore size = 0.015 cm^3^ g^−1^ and pore diameter = 23.35 nm), as summarized in Figure 6b–e [131].

#### 3.3.3. Oxalate Co-Precipitation Route

Oxalate co-precipitation uses oxalic acid, instead of LiOH or NaOH, as the precipitating agent to obtain the NCM precursor, and oxalic acid as the precipitating agent is cheaper and more environmentally friendly than inorganic bases [126]. Briefly, the reaction scheme showed that metal ions from the spent NCM were being chelated with oxalate ion as a first stage; this was followed by ion exchange with the carbonate ion to release Li_2_CO_3_ [119]. Carbonate co-precipitation and oxalate co-precipitation are more stable and more controllable than hydroxide co-precipitation, but carbonate co-precipitation can easily cause segregation of components during the synthesis process, and the uniformity of oxide element distribution cannot be guaranteed. Therefore, oxalate co-precipitation can make the various components in the solution precipitate according to the stoichiometric ratio [126]. Refly et al. [116] demonstrated that LiNi_1/3_Co_1/3_Mn_1/3_O_2_ (NCM) cathode material can be successfully regenerated from end-of-life LIBs through a simple, fast, and environmentally friendly oxalic acid co-precipitation process. The samples can still maintain excellent electrochemical performance, with a specific discharge capacity of 150.6 mAh/g and a capacity retention rate of 91.3% after 100 charge–discharge cycles. Gao et al. [132] systematically studied the influence of reaction pH value, reaction time and reaction temperature on oxalic acid co-precipitation, and found that the best precipitation efficiency can be reached when the co-precipitation is carried out at a temperature of 50 °C, and the pH value of the reaction solution was 1.98. The process was precisely controlled by adding ammonia water during the process. In order to achieve simultaneous recovery of lithium and direct regeneration of cathode materials, Liu et al. [127] used P227 extractant to extract and separate Li directly from transition metals; this was followed by regeneration of the spherical cathode material (LiNi_1/3_Co_1/3_Mn_1/3_O_2_ (NCM111)) by oxalic acid co-precipitation at 55 °C; the material exhibited excellent charge/discharge capacity (182.4 mAh g^−1^/151.0 mAh g^−1^ at 0.5 C) and cycling performance (98% stability after 20 operation cycles).

In summary, based-on the criteria of the NCM precursor co-precipitation scheme illustrated in Figure 4, with selected review examples in this section, even though the use of oxalate co-precipitation produced the best-quality regenerated NCM for the second life LIBs among the three methods (hydroxide, carbonate, and oxalate), due to the relatively green nature of the reaction, the relatively low yield of the co-precipitation product is the main barrier [128] to applying such precipitation reaction in industrial-scale production in the long term. This relatively strengthens the advantage of cost effectiveness shown by the conventional hydroxide co-precipitation method, when applying the technique to industrial-scale reactions. So, hydroxide co-precipitation was still regarded as the best method for the regenerated NCM precursor production.

## 4. Industrial Applications of Hydrometallurgy-Based LIB Recycling

Based on the extensive batch-scaled hydrometallurgy-driven LIB metal recycling research in previous sections, the application of this approach in industrial-scale production lines is the ultimate goal, in order to achieve the goal of metal resource recycling in the chemical supply chain. There are several enterprises focused on hydrometallurgy-driven metal recycling from spent LIBs worldwide, including Umicore (Belgium) [133] and Guanma machinery (PRC) [134] which were set up in 2004 and 2019, respectively. Recently, the Hong Kong-based enterprise of Chun Yang Int Ltd-HK [135] set up a spent-LIB black powder recycling plant at EcoPark HK in early 2026. The mentioned recycling enterprises applied sulfuric acid (2–4 M)/H_2_O_2_ reducing agent-driven hydrometallurgy to recycle valuable metals (Ni, Co, Mn, and Li) from spent LIBs, with an efficiency range of 95–97%. This operationalizes a production line example, as illustrated on its webpage, which consist of a spent battery scrapping line, sulfuric acid-driven hydrometallurgy reaction tanks with filters, and a co-precipitation system for regenerated NCM production on the scale of tons [136]. Recovered metal ions in the mentioned process were converted into individual metal-based salts (e.g., NiSO_4_, CoSO_4_, MnSO_4_, and Li_2_CO_3_) for the chemical supply chain or regenerated ternary NCM cathodic materials for second life LIB applications (e.g., electric devices, EV).

## 5. Challenges and Perspective

To date, metal ions (Li, Ni, Co, and Mn) have been simultaneously recovered from spent-LIB cathode black powder in acid-assisted hydrometallurgy, as reviewed previously. This is associated with a number of challenges. First of all, ineffective Li recovery due to the selectivity towards Li^+^ was the greatest challenge in terms of the leaching efficiency (e.g., negligible Li recovery from 3% LIB recovery in 2015 [137], high energy consumption with low efficiency [138], serious loss of Li [139], and low or even no selectivity towards Li [140]). Furthermore, the natural abundance of Li^+^ was also dramatically reduced [137] due to over-extraction from water sources (aquatic and underground water in soil). The above-mentioned shortage in Li recovery work became another form of resource wasting, especially when applying conventional hydrometallurgy in industrial-scale LIB recycling (metal recovery and chemical reaction synthesized regenerated NCM powder for second life battery production). This would violate the main direction of green, and even sustainable, battery manufacturing in the future.

In addition to the problem of difficult Li recovery using conventional processes, the second challenge is the chemicals being used in conventional hydrometallurgy. The use of conventional mineral acid and organic acid is still associated with problem of secondary pollution due to their acidity and the evolution of toxic pollutants, as mentioned in previous sections [9,23,59]. Furthermore, the reducing agent being used in conventional hydrometallurgy also results in secondary pollution due to residual reducing agent release in water streams upon discharge. A conventional reducing agent like H_2_O_2_ is unstable at room temperature, which results in poor metal recycling efficiency [54,141,142,143,144,145,146]. In order to solve the mentioned problems, alternative reagents were developed, including deep-eutectic solvent (DES) and a biomass-based reducing agent. This is particularly important for the industrialization of hydrometallurgy-driven LIB recycling and in fulfilling the trend towards green approaches and sustainability with low cost. Huge amounts of effort have been invested in fighting against the previously mentioned two challenges.

### 5.1. Selective Lithium Pre-Extraction as Solution to Enhanced LIB Recycling

In order to solve the problem of difficult Li recovery in conventional acid leaching, selective lithium pre-extraction was then proposed as one of directions to prevent unsatisfactory Li recovery in conventional hydrometallurgy. One of the proposed ways is sequential pyrolysis followed by water leaching, as demonstrated by some groups [138,147,148]. Black powder (622, 523, 333) has been roasted under a high temperature (500–1000 °C) with sulphate salts as the first first step (pyrolysis) in producing water-soluble lithium salt via sulphite salt (Na_2_SO_3_) roasting [138] or a modified carbothermic reduction (Li_2_CO_3_ + graphite) [147,148], with the details of the proposed reaction mechanisms as shown in the original texts of the articles [138,147,148]. The roasted products were then treated by water leaching in an ultrasonic bath under a mild temperature (<100 °C) with effective thermal isolation [148]. The Li recovery percentage achieved by this method was very high (95.8 wt% [147], 93–94.06% [138,148]) in terms of high-purity Li_2_CO_3_ produced [138,148]. The residual formed was beneficial for effective Ni, Co, Mn recovery by water- or conventional mineral acid (2 M HNO_3_)-driven hydrometallurgy in terms of high leaching efficiency (Ni: 97.67–99.63%, Co: 96.96–99.84%, Mn: 99.22–99.79%) [138,148]. A regenerated 622 battery even showed high crystallinity and excellent initial discharge capacity (173.8 mAh g^−1^, 0.1 C), cycling performance (157 decreased to 149 mAh g^−1^ at 1 C after 100 cycles), and stability (retention rate of 94.90% after 100 cycles) with the help of the closed-loop recycling system developed by Yang’s group, which consists of roasting and a water leaching system [138]. Such results are indications for the further development of green and effective metal recycling, or even NCM regeneration from spent LIBs for the purposes of industrialization.

### 5.2. Use of Alternative Reagents for Sustainable Hydrometallurgy-Driven Spent-LIB Metal Recycling

DES is an ionic solvent composed of a hydrogen bond acceptor (HBA) (choline chlorine [149,150,151] or tri-octylphosphine oxide (TOPO) [152]) and organic compounds (urea [153,154], oxalic acid [153], citric acid [149], ethylene glycol (EG) [150,153] p-toluenesulphonic acid (PTSA) [151], decanoic acid [152], etc.) as hydrogen bond donor (HBD). They act as the chelating group for effective capturing of the precious metal ion (e.g., Co) from spent-LIB black powder via chelate bond formation between Li, Co (LCO in spent LIBs) and an oxygen atom (DES) [149]. Such solvents have the strong advantages of low cost and biodegradability, which has made them the new star leaching agents for metal recovery from spent LIBs [149,150,151,153]. The use of such a solvent has the advantage of neutral pH with high leaching efficiency towards different LIB metals, while the leaching temperature can be mild (~80 °C) to as high as 180 °C, as summarized in Table 5. Demonstration of EG:ChCl DES-driven metal recovery from spent LCO and NCM LIBs by Tran’s group showed the high Co (LCO: 50.30%; NCM: 31.71%) and Li (LCO: 89.91%; NCM: 70.80%) leaching efficiency [150]. EG:ChCl DES can be reused via recycling by an electrodeposition of Co from the Co^2+^/EG:ChCl mixture (LCO leachate), and performance of recovered EG:ChCl DES was maintained in second round of LCO leaching [150] Further improvement of the DES-driven hydrometallurgy is expected in order to make the process a more green (mild leaching temperature, recyclable DES, etc.) reaction with high leaching efficiency (~99%) when spent-LIB black-mass mixture is used and the process is upscaled to the industrial level in the future.

**Figure 7:**
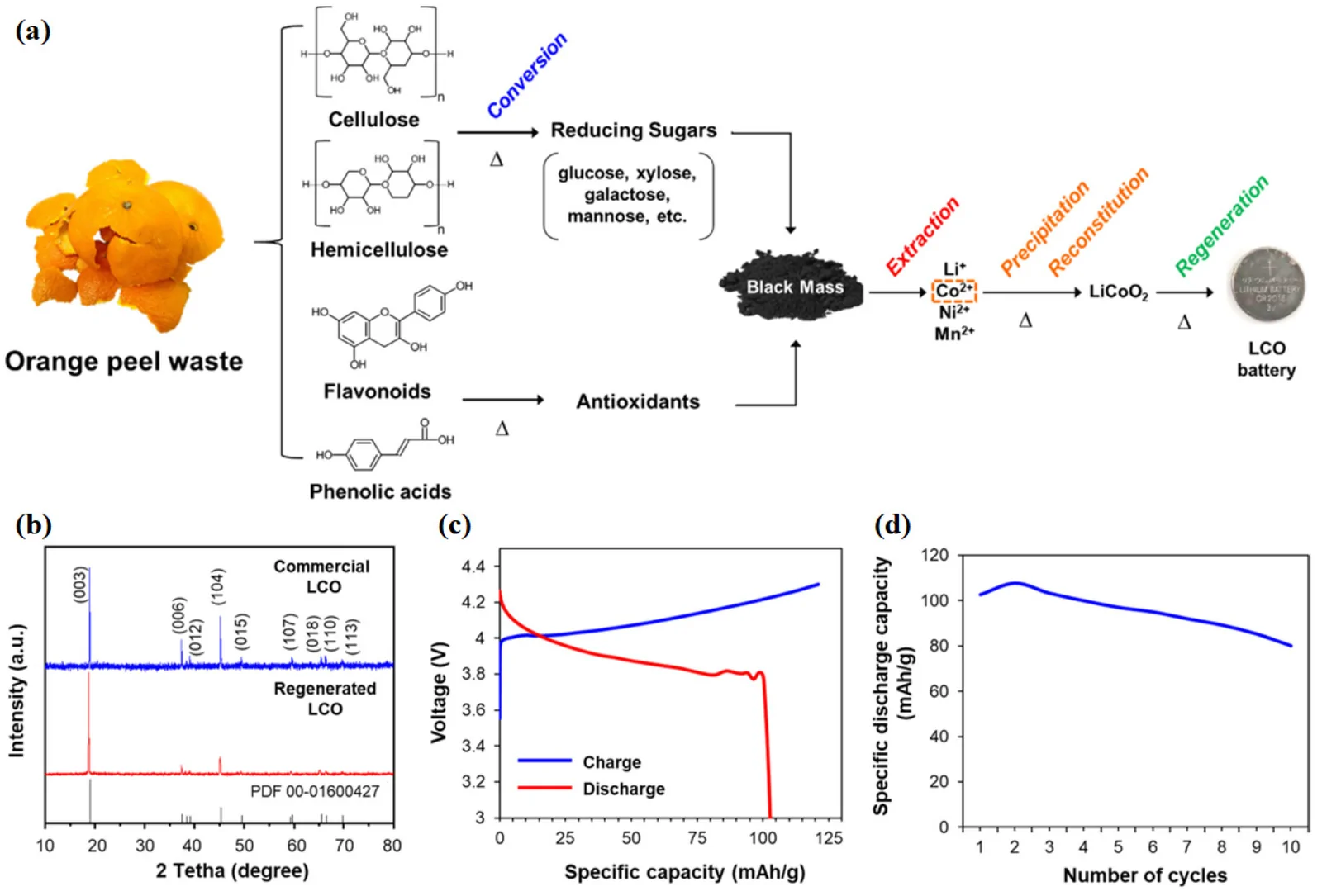
(**a**) Mechanistic and process summary of OP-enabled recycling of spent LIBs. (**b**) XRD pattern of the commercial (blue) and regenerated (red) LCO. (**c**) Initial charge (blue)−discharge (red) performance of the regenerated LCO coin cell and (**d**) stability of discharge capacity over 10 cycles of charge and discharge [142]. (Reproduced with permission, 2020, ACS publication).

As to the case of a biomass-based alternative reducing agent, vegetable fiber is major source; this is summarized in Table 6. Since carbohydrates [54,142,143,155], cellulose [155], ethanol [51], phenol [143,144], aldehyde [144], catechin [141], epicatechin (EC) [141], epigallocatechin gallate (EGCG) [141], and so on in the vegetable-base food residual each exhibited strong reducing power in precious metal recovery. To date, the demonstrations summarized in Table 7 have shown that metal recovery (Li, Co) from spent LIBs was 92–96% when using biomass reducing agent, which is comparable to the conventional inorganic reducing agents H_2_O_2_ and sodium sulfites. A demonstration of citric acid-driven valuable metal recovery (Li, Ni, Co, Mn) from black mass spent-LIB mixture performed by Wu’s group showed that reducing agent from orange peel (cellulose) exhibited strong Li (80.5%), Ni (90.1%), Co (91.3%), and Mn (92.2%) recovery (Table 7) with the mechanism illustrated in Figure 7a [142]. Cell performance of regenerated LCO-fabricated LIBs from Co extracted via precipitation (spent-LIB black mass) showed initial capacity comparable to the commercial LCO-type LIBs (charging: 120 mAhg^−1^; discharging: 103 mAhg^−1^) with stable performance (20% capacity loss) after 10 cycles charging/discharging operation, and high crystallinity matching with commercial LCO from XRD analysis (Figure 7b–d) [142]. However, the active reducing ingredients in the biomass are low in total amount (<2%) which made their application in the effective hydrometallurgy (99% or above) still challenging [144,156]. As a result, careful optimization will be the solution in developing low-cost, highly effective, and environmentally friendly acid-assisted hydrometallurgy in future.

As a perspective on the implementation of hydrometallurgy development for industrial-scale recycling in the future, implementing selective lithium pre-extraction as part of the spent-LIB metal recycling process and using alternative green chemicals with biomass-based reducing agents in the hydrometallurgy process or production line is one direction. Also, a certain degree of automation may be required in order to control the use of chemicals accurately in order to prevent potential secondary pollution due to residual reagent discharge to water and air streams throughout the hydrometallurgy-based process. For example, the implementation of machine learning, as proposed by Hu et al. [157], emphasizes optimizing the combination of the input of raw materials (NCM, LFP, and LCO) and leaching reaction conditions (leachant candidates, operation parameters) to simulate the predicted results via ANOVA and simulation modelling for the final products produced. This way can be regarded as one of the ways to minimize energy consumption and wastage of residual chemicals throughout the hydrometallurgy, especially in a potential industrial-scale operation.

## 6. Conclusions and Forecast

In conclusion, hydrometallurgy is regarded as one of the green and effective ways to recycle valuable metals from spent LIBs in order to achieve the green economy, especially the recycling of valuable metals from cathodes of LIBs. The use of an acid with a reducing agent to extract metal ions, followed by purification via separation and precipitation is the most extensively implemented method used to generate second life metal products or regenerated batteries. Metal leaching processes have involved the use of mineral acid or organic acid with organic or inorganic reducing agents, which has achieved a high leaching efficiency of up to 90%, or even complete recycling. The valuable metals in the leaching solution are usually recovered by chemical co-precipitation or extraction with purification by either physical membrane filtration or solvent extraction. The solvent extraction method is more efficient, while the use of a membrane can make the process easy to control and reduce the amounts of chemicals used. The chemical co-precipitation method can directly synthesize valuable metals into the cathode materials required for batteries in one step while ensuring recovery efficiency.

However, this is a double-edged sword for hydrometallurgy. First of all, the use of an organic acid is usually associated with relatively low reaction kinetics or low recycling efficiency, while mineral acids usually result in secondary pollution due to the evolution of toxic gas and require high acid concentration as a driving force. The instability of inorganic reducing agents like H_2_O_2_ and sulfite-based salts are associated with efficiency reduction. Secondly, conventional solvent extraction cannot extract multiple valuable metals at the same time. Filtration is incapable of filtering out metallic impurities of the same valency in the leachate mixture. Thirdly, the recovery of Li^+^ by conventional chemical precipitation and hydrometallurgy is unsatisfactory. Fourthly, most of the hydrometallurgy-based LIB recycling research to date has focused on a single spent battery source and the regenerated final NCM product was single-ratio only. It cannot reflect the real situation involving the mixture of received spent batteries. All of the above-mentioned challenges are barriers to developing green, industrial-scale spent-LIB recycling. To unleash the potential and strength of hydrometallurgy, first of all, the extraction method and the chemical precipitation method should be used in combination. After the specific recyclable metals are extracted by the extraction method, the remaining metal ions are co-precipitated. Secondly, selective lithium pre-extraction with low energy consumption and high Li leaching efficiency (>90%) can be separated from NCM-only residual and become the solution to conventional hydrometallurgy-assisted spent-LIB recovery. Thirdly, hydrometallurgy of the spent-LIB mixture and regenerated final NCM product synthesis with tailorable NCM ratio should be the future research direction in order to reflect the situation of battery recycling plant operation as a potential upgrade to the hydrometallurgy process as a pilot scheme or even in industrial scale production for sustainable battery manufacturing. Fourthly, use of DES and a biomass-based reducing agent will be another solution fighting against the secondary pollution and low efficiency due to conventional reducing agents and the acidic leaching agents being used in conventional hydrometallurgy. Finally, optimization of the process should also be assisted with machine learning to a certain extent. All of these proposed ways will be beneficial to future hydrometallurgy-based LIB recovery in a green and sustainable way, especially with the shift to industrial scale while using the best approach.

## Figures and Tables

**Table 3:** List of activation energy values of acid leachants, and corresponding kinetic model.

Leachant	Leaching Kinetic Model	Activation Energies E_a_ (kJ/mol)				Ref.
		Li	Ni	Co	Mn	
Oxalic acid	Logarithmic model	9.7	11.2	9.5	6.2	[86]
Benzenesulfonic acid	Shrinking core model	41.06	N/A	35.21	N/A	[59]
Trichloroacetic acid	Avrami equation	28	44.51	44.79	43.81	[75]
Tartaric acid	Shrinking core model	54.03	73.28	58.18	66	[66]
Tartaric acid	Shrinking core model	53.86	47.78	58.94	55.68	[66]
Phosphoric acid	Logarithmic model	10.2	N/A	7.3	N/A	[46]
Sulfuric acid	Shrinking core model	N/A	29.35	24.00	23.29	[87]
Malic acid	Avrami equation	41.71	42.83	44.38	43.17	[83]

N/A: Data not available.

**Table 4:** Best metal rejection percentages and separation factors (lithium to multivalent element) by NF membranes.

Spent LIB, Black Powder Type	Membrane	Rejection (%)	Separation Factor	Ref.
NCM	NF1000-EDA	N/A	645.9 (Ni), 508.8 (Co), 307.4 (Mn)	[112]
LCO	PA-M2	Li = 64.8%, Co = 99.9%	351.5 (Co)	[113]
LFP	VNF2	Ni = 92.5%, Co = 94.6%, Mn = 95.8%	N/A	[114]
NCM simulating solution	Desal DL	Li = 1.5%, Ni = 96.4%, Co = 96.1%, Mn = 94.4%, Al = 98.6%, Cu = 47.0%, Fe = 94.1%	4.03	[115]
NCM simulating solution	Desal KH	Li = 18.0%, Ni = 63.3%, Co = 63.1%, Mn = 62.0%, Al = 88.2%, Cu = 53.6%, Fe = 94.1%	2.11	[115]

N/A: No data available.

**Table 5:** Best metal recovery efficiency of DES agent-driven hydrometallurgy.

Spent LIB, Black Powder Type	DES (Mole Ratio)	Temperature (°C) and Time (h)	Efficiency (%)	Ref.
LiCoO_2_	Citric acid:ChCl (1:2)	80 °C, 1 h	Li = 96.98%, Co = 94.26%	[149]
LIBs black powder mixture	Decanoic acid:TOPO (1:1)	80 °C, 4 h	Co = 96%, Mn = 91%	[152]
Pure LiMn_2_O_4_	Oxalic acid:ChCl (1:1)	75 °C, 15 min	Li = 99%, Mn = 96%	[153]
LiMn_2_O_4_ powder from spent battery	Oxalic acid:ChCl (1:1)	75 °C, 15 min	Li = 99%, Mn = 95%	[153]
LiCoO_2_	PTSA:H_2_O:ChCl (1:2:1)	90 °C, 15 min	Li = 100%, Co = 100%	[151]
LiCoO_2_	Urea:ChCl (2:1)	180 °C, 12 h	Li = 94.7%, Co = 97.9%	[154]
LiCoO_2_	Ethylene glycol:ChCl (2:1)	180 °C, 24 h	Li = 89.91%, Co = 50.30%	[150]
NCM (333)	Ethylene glycol:ChCl (2:1)	180 °C, 24 h	Li = 70.08%, Ni = 6.65%, Co = 31.71%, Mn = 60.16%	[150]

**Table 6:** Reductive ingredients in biomass materials.

Biomass	Active Reducing Agent	Ref.
Waste areca	Cellulose, lignin, phenol	[144]
Glucose	Glucose	[54,155]
Sucrose	Sucrose	[155]
Cellulose	Cellulose	[155]
Ethanol	Ethanol	[51]
Orange peel	Cellulose, glucose	[142]
Tea	Catechin, epicatechin, epigallocatechin gallate	[143]
Grape seed	Catechin, epicatechin, epigallocatechin gallate	[141]

N/A: No data available.

**Table 7:** Best metal recovery efficiency of biomass reducing agent-driven hydrometallurgy.

Spent LIB, Black Powder Type	Acid (Solid to Liquid Ratio)	Reducing Agent (Dosage g/g)	Temperature (°C) and Time (h)	Efficiency (%)	Ref.
NCM622	H_2_SO_4_ (3 M, 20 mL/g)	Waste areca:NCM = 1:1	90 °C, 2 h	Li = 99.9%, Ni = 99.9%, Co = 99.9%, Mn = 99.8%	[144]
LiCoO_2_	H_2_SO_4_ (3 M, 25 g/L)	Glucose:LCO = 0.4 g/g	95 °C, 2 h	Li = 96%, Co = 98%	[155]
LiCoO_2_	H_2_SO_4_ (3 M, 25 g/L)	Sucrose:LCO = 0.4 g/g	95 °C, 2 h	Li = 100%, Co = 96%	[155]
LiCoO_2_	H_2_SO_4_ (3 M, 25 g/L)	Cellulose:LCO = 0.4 g/g	95 °C, 2 h	Li = 100%, Co = 54%	[155]
LiCoO_2_	H_2_SO_4_ (3 M, 20 g/L)	Ethanol (5% *v*/*v*):LCO = N/A	90 °C, 2⅔ h	Li = 99%, Co = 99%	[51]
LiCoO_2_	H_3_PO_4_ (1.5 M, 20 g/L)	Glucose (0.02 M):LCO = N/A	80 °C, 2 h	Li = 99.75%, Co = 98.01%	[54]
LiCoO_2_, NCM mixture	Citric acid (1.5 M, 25 g/mL)	Orange peel:spent cathode powder = 250 mg:200 mg	100 °C, 4 h	Li = 80.5%, Ni = 90.1%, Co = 91.3%, Mn = 92.2%	[142]
LiB cathode black powder mixture	H_2_SO_4_ (2 M, 50 g/L)	Tea:Black powder = 200 mg:250 mg	100 °C, 4 h	Li = 90.7%, Ni = 92.1%, Co = 57.3%, Mn = 84.6%	[143]
LiCoO_2_	Malic acid (2 M, 20 g/L)	Grape seed:LCO = 0.6 g/g	80 °C, 3 h	Li = 98.77%, Co = 92.13%	[141]

N/A: No data available.

## Data Availability

The original contributions presented in this study are included in the article/Supplementary Material. Further inquiries can be directed to the corresponding author.

## Supplementary Materials

The following supporting information can be downloaded at: https://www.mdpi.com/article/10.3390/ma19194242/s1, Figure S1. Yearly publications on LIB recycling: (a) graphical and (b) table format and (c) list of countries with at least 1% publication on LIB recycling. Figure S2. Effect on leaching efficiency of (a) initial acid concentration, (b) solid/liquid ratio, (c) temperature, (d) amount of H_2_O_2_, (e) reaction time, and (f) metal leaching efficiencies under optimized conditions. Figure S3. Schematic illustration of the reaction mechanism in the mechanochemical process. Figure S4. (a) Possible reaction products when using L-tartaric acid as a leachant. Effect of (b) H_2_O_2_, (c) L-tartaric acid concentration, and (d) pulp density on the leaching efficiencies of Mn, Li, Co, and Ni (17 g/L solid-to-liquid ratio (S/L), 70 °C, and 30 min). Figure S5. Effect of various parameters (a: time, b: temperature, c: acid concentration, d: solid to liquid ratio) on the leaching efficiencies of metal ions. (e) Flowsheet of the regeneration processes of NCM cathodes from spent LIBs. Figure S6. Selected commonly used solvent for liquid-liquid extraction of metal ions from hydrometallurgy leachates. Figure S7. Proposed flowchart for recovering Li, Co, Ni, and Mn from the leachate via solvent extraction. Figure S8. Schematic diagrams of the conventional and membrane recycling methods. Figure S9. Schematic illustration of surface distribution for unmodified and EDA-modified membranes under neutral and acidic conditions. Figure S10. Schematic Drawing of Formation Process of Microscale Core-Shell Structured Li[(Ni_0.8_Co_0.1_Mn_0.1_)1−x(Ni_0.5_Mn_0.5_)x]O_2_. Figure S11. Cycling profile of (a) R333, (b) R523, (c) R532, and (d) R622 CR2032 battery in the first 50 cycles at 0.1 C scan rate.
