# Supplementary material for: Recycling and Recovery of Cathode Materials from Spent Lithium-Ion Batteries by Hydrometallurgy: A Review
Source: Materials (Basel). 2026 Oct 7;19(19):4242. doi: 10.3390/ma19194242 (PMC13666252; doi:10.3390/ma19194242)
Supplement: Supplementary file 1 [file materials-19-04242-s001.zip › materials-4334881-supplementary.pdf]

materials

MDPI

Review

Recycling and Recovery of Cathode Materials from Spent Lithium-Ion Batteries by Hydrometallurgy: A Review

Alpha Chi Him Tsang, Jing Ji, Dawson Wai-Shun Suen, Chi-Wing Tsang, Vanessa Chi Ying Li and Xiao-Ying Lu *

Department of Construction, Environment and Engineering, Technological and Higher Education Institute of Hong Kong; 20A, Tsing Yi Road, Tsing Yi, Hong Kong; achtsang@thei.edu.hk (A.C.H.T.); hku507@gmail.com (J.J.); dawsonsuen@thei.edu.hk (D.W.-S.S.); ctsang@thei.edu.hk (C.-W.T.); cyvli@thei.edu.hk (V.C.Y.L.)
* Correspondence: xylu@thei.edu.hk

Supplementary Information

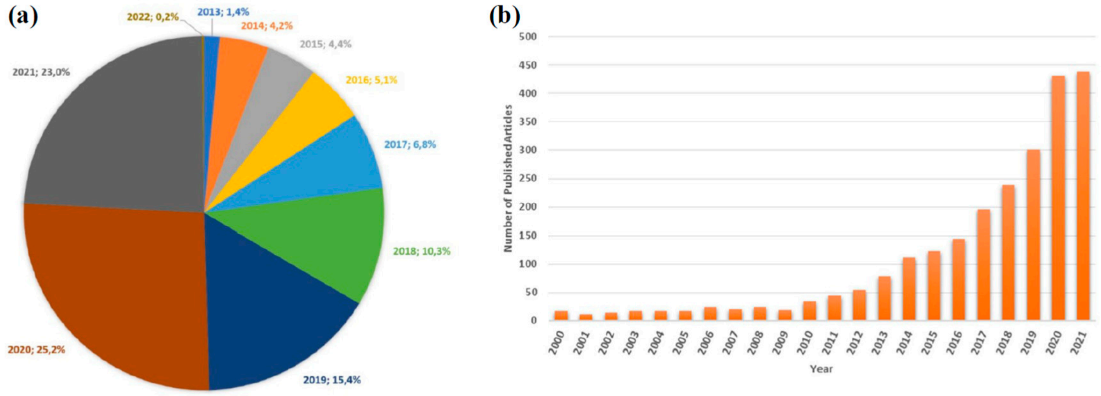

(c)

Countries	Number of Publications	Percentage (%)
China	992	40.2
USA	312	12.6
South Korea	195	7.9
Japan	153	6.2
Germany	142	5.8
India	132	5.3
Australia	121	4.9
Canada	87	3.5
England	79	3.2
France	67	2.7
Italy	55	2.2
Iran	52	2.1
Finland	48	1.9
South Africa	48	1.9
Brazil	47	1.9
Spain	47	1.9
Sweden	40	1.6
Singapore	33	1.3
Taiwan	33	1.3
Belgium	29	1.2
Turkey	25	1.0

Figure S1. Yearly publications on LIB recycling: (a) graphical and (b) table format and (c) list of countries with at least 1% publication on LIB recycling. [1] (Reproduced with permission, 2022, MDPI).

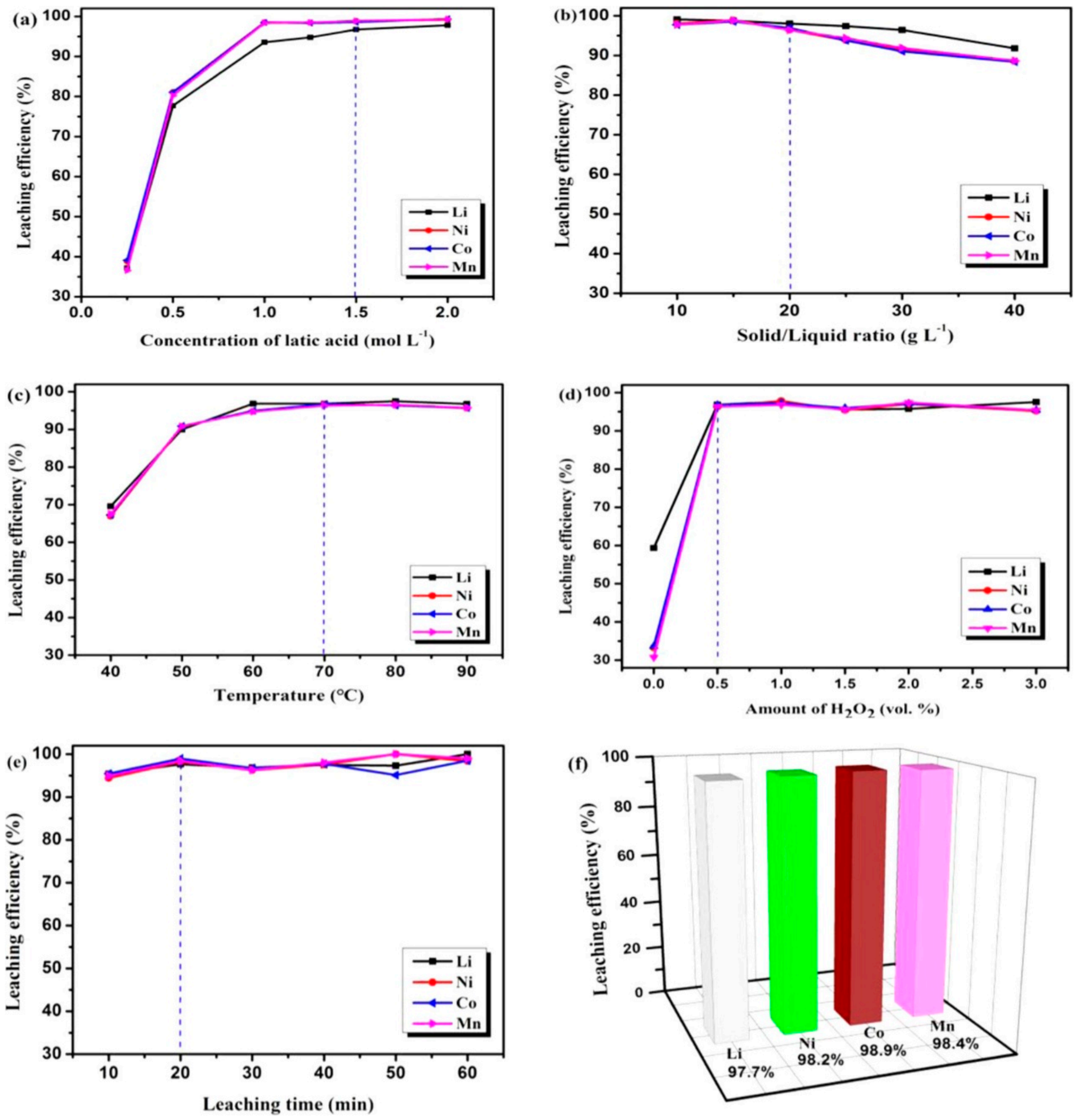

Figure S2. Effect on leaching efficiency of (a) initial acid concentration, (b) solid/liquid ratio, (c) temperature, (d) amount of H_2O_2, (e) reaction time, and (f) metal leaching efficiencies under optimized conditions [2]. (Reproduced with permission, 2017, ACS Publications).

https://doi.org/10.3390/ma19194242

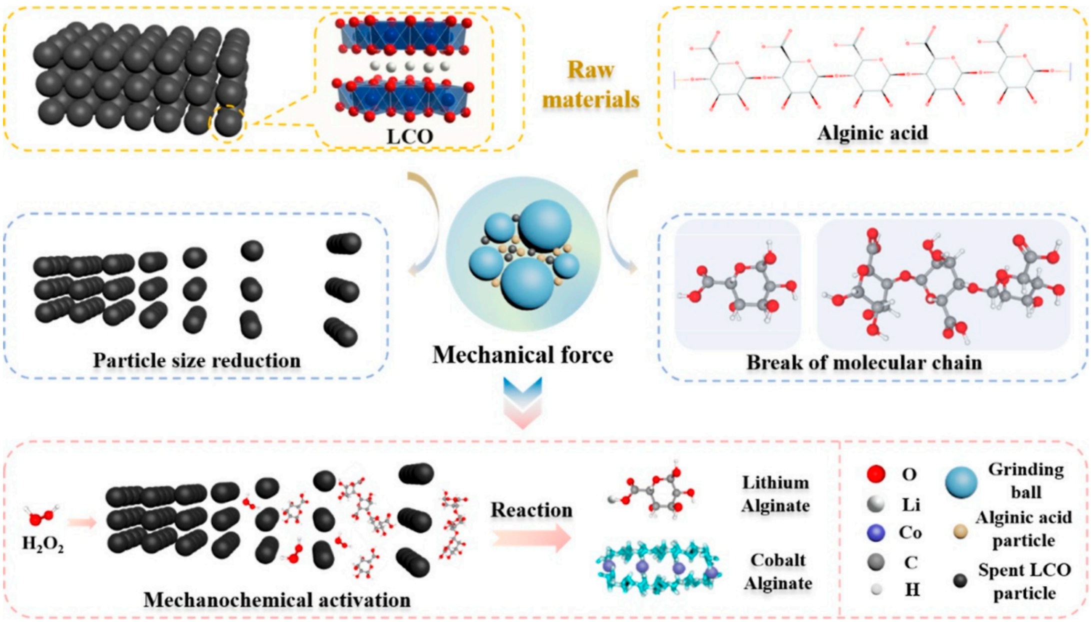

Figure S3. Schematic illustration of the reaction mechanism in the mechanochemical process [3]. (Reproduced with permission, 2021, ACS Publications).

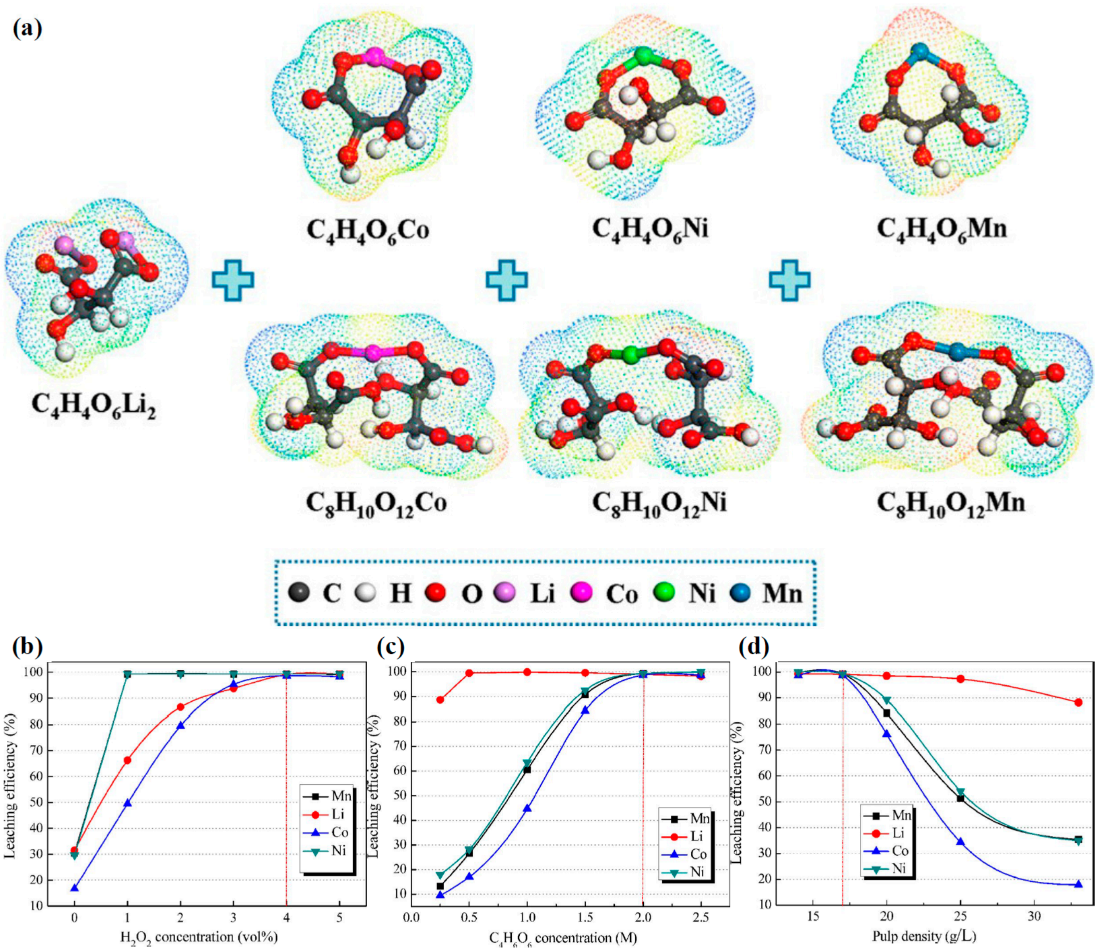

Figure S4. (a) Possible reaction products when using L-tartaric acid as a leachant. Effect of (b) H_2O_2, (c) L-tartaric acid concentration, and (d) pulp density on the leaching efficiencies of Mn, Li, Co, and Ni (17 g/L solid-to-liquid ratio (S/L), 70 °C, and 30 min) [4]. (Reproduced with permission, 2017, ACS Publications).

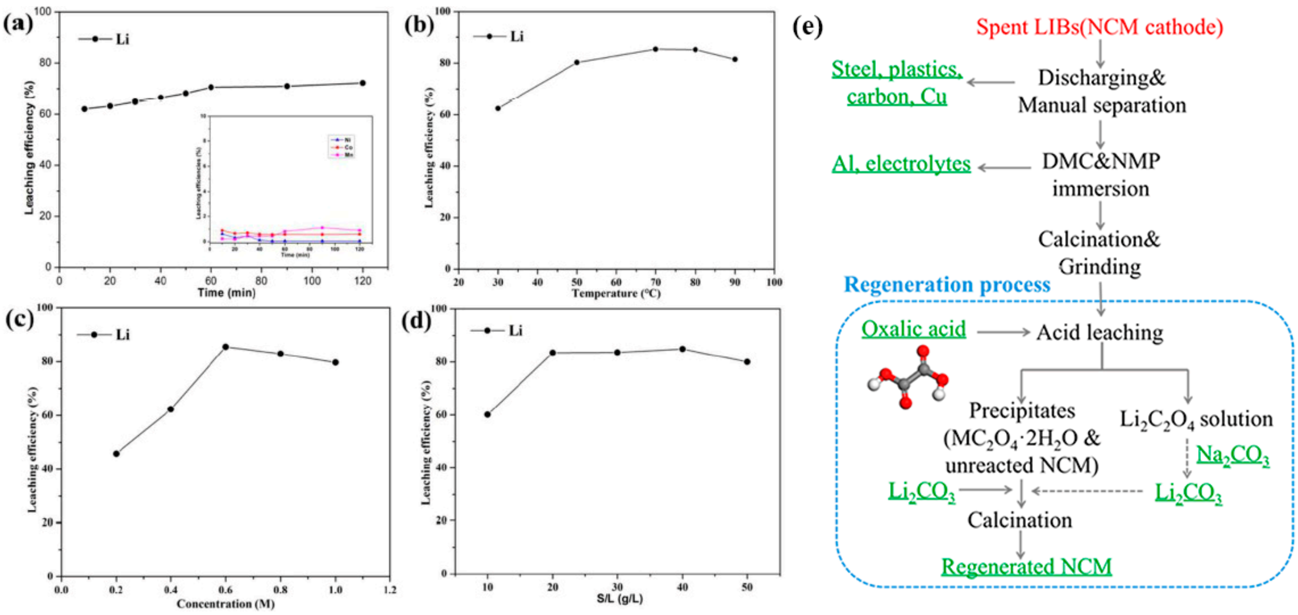

Figure S5. Effect of various parameters (a: time, b: temperature, c: acid concentration, d: solid to liquid ratio) on the leaching efficiencies of metal ions. (e) Flowsheet of the regeneration processes of NCM cathodes from spent LIBs [5]. (Reproduce with permission, 2018, ACS Publication).

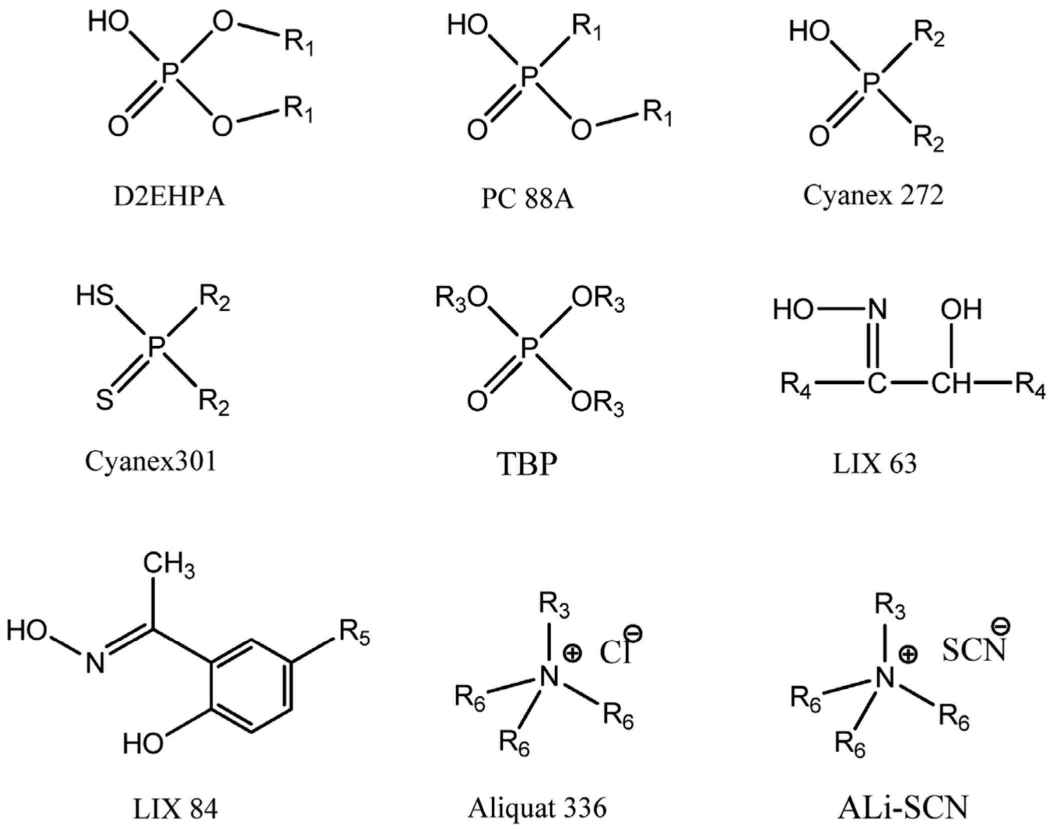

*Denote: R_1, R_2, R_3, R_4, R_5, R_6 are 2-ethylhexyl-, 2,4,4-trimetylpentyl-, n-butyl-, 1-ethylpentyl-, n-nonyl-, n-octyl- groups, respectively.

Figure S6. Selected commonly used solvent for liquid-liquid extraction of metal ions from **hydrometallurgy** leachates. [6] (Reproduced with permission, 2021, Wiley VCH-GmbH & Society of Chemical Industry (SCI)).

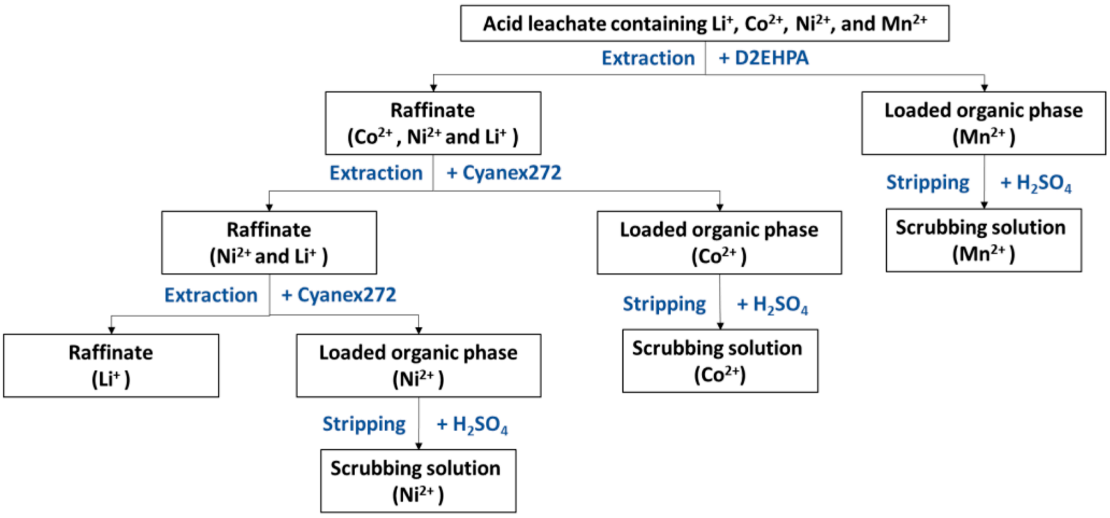

Figure S7. Proposed flowchart for recovering Li, Co, Ni, and Mn from the leachate via solvent extraction. [7] (Reproduced with permission, 2023, MDPI).

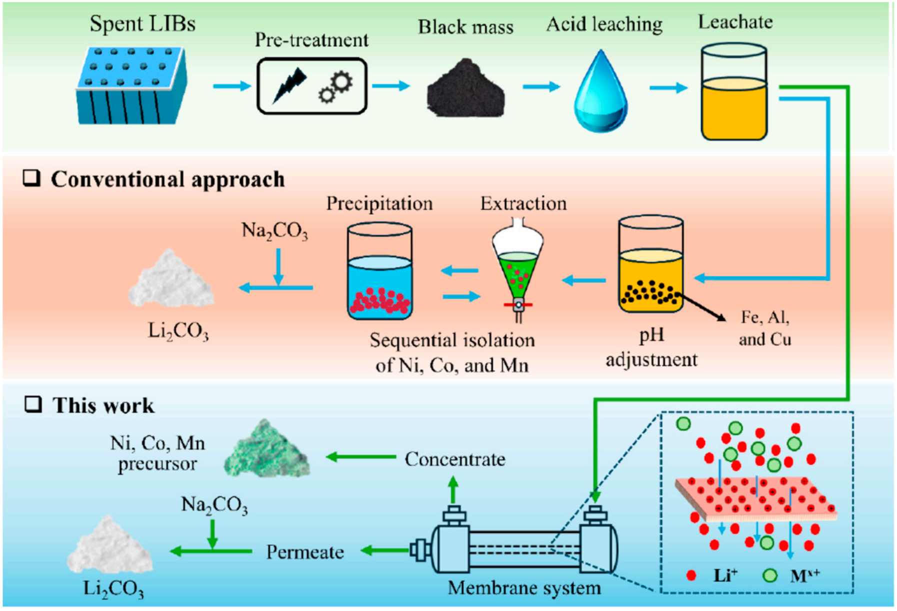

Figure S8. Schematic diagrams of the conventional and membrane recycling methods. [8] (Reproduce with permission, 2025, ACS Publications).

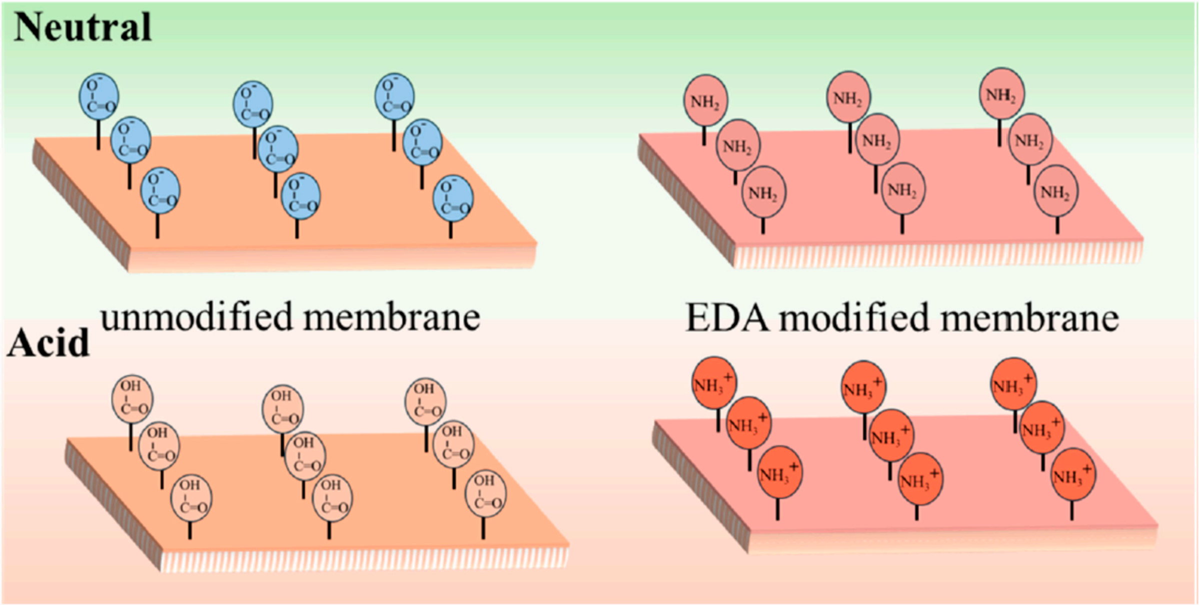

Figure S9. Schematic illustration of surface distribution for unmodified and EDA-modified membranes under neutral and acidic conditions. [8] (Reproduce with permission, 2025, ACS Publications).

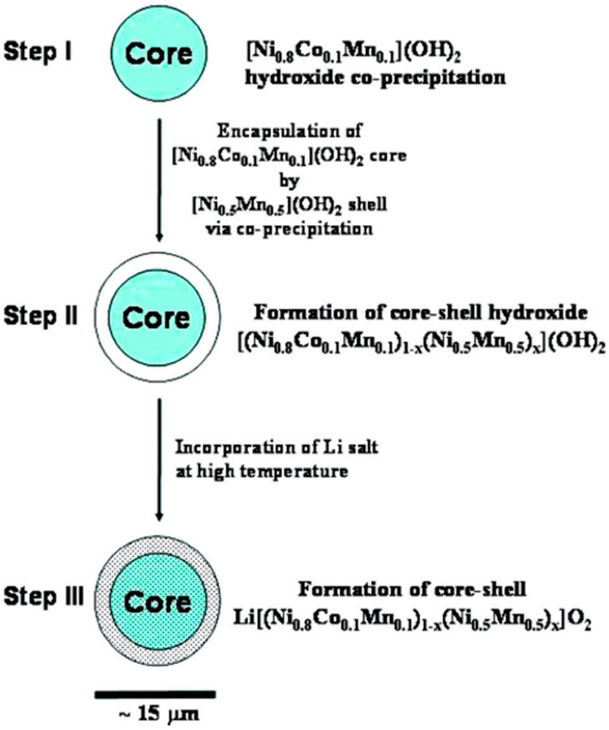

Figure S10. Schematic Drawing of Formation Process of Microscale Core-Shell Structured $Li[(Ni_{0.8}Co_{0.1}Mn_{0.1})_{1-x}(Ni_{0.5}Mn_{0.5})_x]O_2$. [9] (Reproduced with permission, 2005, ACS publication).

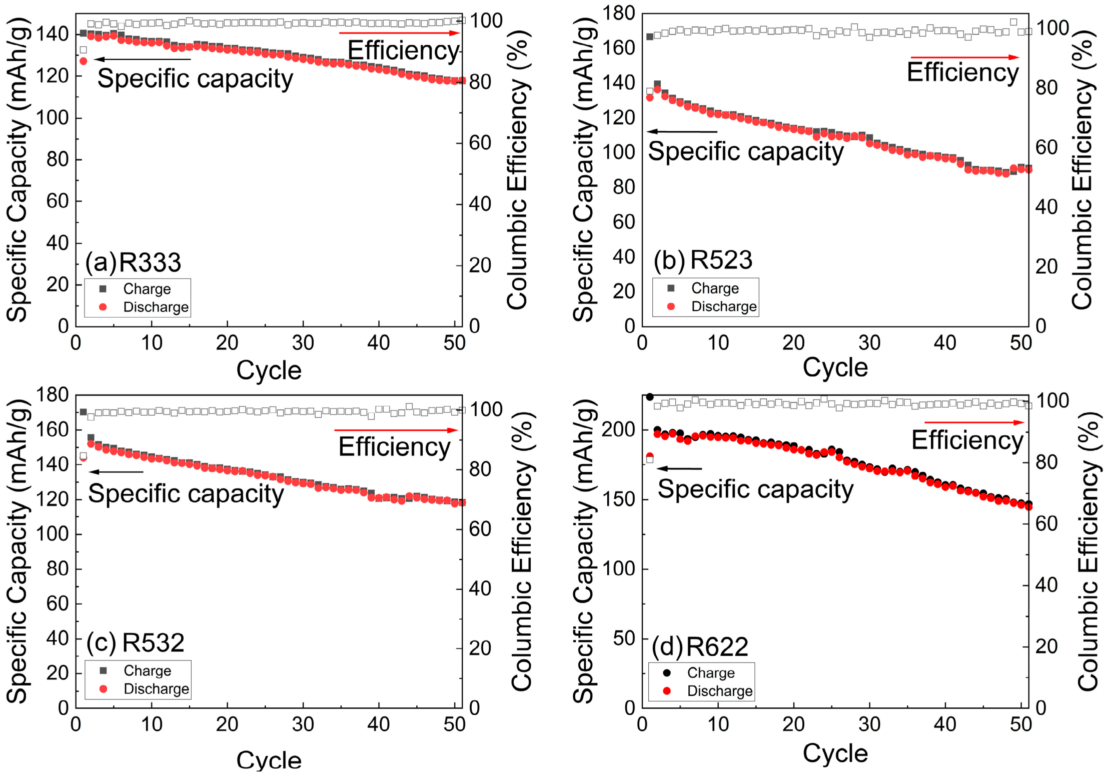

Figure S11. Cycling profile of (a) R333, (b) R523, (c) R532, and (d) R622 CR2032 battery in the first 50 cycles at 0.1 C scan rate. [10] (Reproduced with permission, 2024, MDPI).

References

1. Regatieri, H. R.; Ando Junior, O. H.; Cezar Salgado, J. R., Systematic Review of Lithium-Ion Battery Recycling Literature Using ProKnow-C and Methodi Ordinatio. *Energies* **2022,** 15, (4), 1485.
2. Li, L.; Fan, E.; Guan, Y.; Zhang, X.; Xue, Q.; Wei, L.; Wu, F.; Chen, R., Sustainable Recovery of Cathode Materials from Spent Lithium-Ion Batteries Using Lactic Acid Leaching System. *ACS Sustain. Chem. Eng.* **2017,** 5, (6), 5224-5233.
3. Cai, L.; Lin, J.; Fan, E.; Wu, F.; Chen, R.; Li, L., Eco-Friendly Organic Acid-Assisted Mechanochemical Process for Metal Extraction from Spent Lithium-Ion Batteries. *ACS Sustain. Chem. Eng.* **2022,** 10, (32), 10649-10657.
4. He, L.-P.; Sun, S.-Y.; Mu, Y.-Y.; Song, X.-F.; Yu, J.-G., Recovery of Lithium, Nickel, Cobalt, and Manganese from Spent Lithium-Ion Batteries Using L-Tartaric Acid as a Leachant. *ACS Sustain. Chem. Eng.* **2017,** 5, (1), 714-721.
5. Zhang, X. X.; Bian, Y. F.; Xu, S. W. Y.; Fan, E. S.; Xue, Q.; Guan, Y. B. A.; Wu, F.; Li, L.; Chen, R. J., Innovative Application of Acid Leaching to Regenerate Li(Ni1/3Co1/3Mn1/3)O2 Cathodes from Spent Lithium-Ion Batteries. *ACS Sustain. Chem. Eng.* **2018,** 6, (5), 5959-5968.
6. Tran, T. T.; Moon, H. S.; Lee, M. S., Recovery of valuable metals from the hydrochloric leaching solution of reduction smelted metallic alloys from spent lithium-ion batteries. *Journal of Chemical Technology and Biotechnology* **2022,** 97, (5), 1247-1258.
7. Tang, Y.-C.; Wang, J.-Z.; Shen, Y.-H., Separation of Valuable Metals in The Recycling of Lithium Batteries via Solvent Extraction. *Minerals* **2023,** 13, (2), 285.
8. Yao, X. S.; Zheng, Q. X.; Tjambiru, V. T.; Wu, P. P.; Li, Z. X.; Miyamoto, A.; Watanabe, M., High-Selectivity Separation of Lithium from Spent Lithium-Ion Battery Leachates Using Surface-Modified Commercial Nanofiltration Membranes. *Environmental Science & Technology* **2025,** 59, (43), 23542-23555.
9. Sun, Y. K.; Myung, S. T.; Kim, M. H.; Prakash, J.; Amine, K., Synthesis and characterization of Li[(Ni0.8Co0.1Mn0.1)0.8(Ni0.5Mn0.5)0.2]O2 with the microscale core-shell structure as the positive electrode material for lithium batteries. *J. Am. Chem. Soc.* **2005,** 127, (38), 13411-13418.

10. Tsang, A. C. H.; Ouyang, S.; Lv, Y.; Lee, C. C.; Tsang, C.-W.; Lu, X.-Y., Full Tailored Metal Content NCM Regeneration from Spent Lithium-Ion Battery Mixture Under Mild Condition. *Electrochem* **2024,** 5, (4), 546-559.

Disclaimer/Publisher's Note: The statements, opinions and data contained in all publications are solely those of the individual author(s) and contributor(s) and not of MDPI and/or the editor(s). MDPI and/or the editor(s) disclaim responsibility for any injury to people or property resulting from any ideas, methods, instructions or products referred to in the content.

https://doi.org/10.3390/ma19194242
